# Z-Drugs in the Environment: A Review

**DOI:** 10.3390/molecules31060974

**Published:** 2026-03-13

**Authors:** Anna Topolewska, Aleksandra Zahorska, Agnieszka Łakocka, Jolanta Kumirska

**Affiliations:** Division of Didactics and Popular Science, Faculty of Chemistry, University of Gdansk, Wita Stwosza 63, 80-308 Gdansk, Poland; anna.topolewska@ug.edu.pl (A.T.); aleksandra.zahorska@ug.edu.pl (A.Z.); agnieszka.lakocka@phdstud.ug.edu.pl (A.Ł.)

**Keywords:** z-drugs, zolpidem, zaleplon, zopiclone, eszopiclone, analytical methods, environment, fate, removal in WWTP, ecotoxicity assessment

## Abstract

According to the World Health Organization (WHO), substance dependence and mental health disorders, such as anxiety, depression, post-traumatic stress disorder (PTSD), insomnia, bipolar disorder, and schizophrenia, affect >360 million people worldwide. As a result the increasing use of psychoactive pharmaceuticals, including non-benzodiazepines (also referred to as Z-drugs), has been observed. The COVID-19 pandemic has also had an additional significant negative effect on people’s mental health. Among the aforementioned mental health disorders, chronic insomnia is reported to affect approximately 10% of the adult population. Z-drugs are frequently used in the treatment of insomnia due to their rapid onset of action. They are metabolized in the human organism, but noticeable amounts of the original compound are released to the environment via household wastewater. The extensive use of these pharmaceuticals has led to growing concern about the occurrence of their residues in the environment. Unfortunately, the information on the analytical methods for determining Z-drugs, their main metabolites and transformation products in the environment, efficiency of their removal in wastewater treatment plants, their fate, their presence in environmental matrices, and their ecotoxicological effects is limited. This review paper focuses on summarizing data on these topics. To the best of our knowledge, such a comprehensive review has not yet been published.

## 1. Introduction

Insomnia is a frequently occurring sleep disorder in which individuals struggle to fall asleep, stay asleep, or wake up too early. These symptoms appear on at least three nights each week and cause notable impairment in daytime functioning [1,2,3]. About 10% of adults experience chronic insomnia with a duration of at least 1 month [4,5]. It can lead to various negative outcomes, including missing work, workplace accidents, problems with thinking and concentration, and greater use of healthcare services. It has been shown that insomnia increases with age and is more prevalent in women than in men [6]. Mental health conditions, particularly anxiety and depression, substantially heighten the chance of experiencing insomnia. On the other hand, chronic insomnia substantially raises the likelihood of developing depression, anxiety disorders, substance use disorders, suicidality, hypertension, and diabetes [5,6,7,8].

Sleep problems significantly increased during the COVID-19 pandemic. It is estimated that around 36% of people had symptoms of insomnia during the first wave of COVID-19 [9], and up to 31% of people with long COVID experience disrupted sleep as a long-term issue [10]. Thus, insomnia poses significant challenges to public health [6,7,8,9,10].

Non-benzodiazepine hypnotic drugs (Z-drugs) are among the most frequently prescribed drugs worldwide, used to treat insomnia, with a demonstrated efficacy in treating sleep disorders [11,12,13,14]. Their popularity among patients and clinicians is primarily due to their effectiveness and rapid onset compared to other agents such as antidepressants, which typically require weeks to months before perceived benefit. These substances, like benzodiazepines (BZDs), bind to a specific site on the GABA_A_R receptor (also presented as the GABAA or GABA_A receptor in the literature), enhancing its response to GABA and thereby increasing inhibitory neurotransmission [15]. However, Z-drugs (zopiclone, eszopiclone, zolpidem, zaleplon) exhibit specific affinity for the α1 subunit of the GABA_A_R, resulting in hypnotic effects with reduced anxiolytic and muscle relaxant characteristics when compared to BZDs [15].

Z-drugs were introduced into the market in the 1990s [16]. Though Z-drugs are widely recognized as being effective, they are not without potential harms. Long-term use of Z-drugs has been associated with major adverse events, including—but not limited to—falls and fractures, domestic and traffic accidents, confusion, cognitive impairment, Alzheimer’s disease, and cancer. Various adverse events resulting from, or associated with Z-drugs, have been presented and discussed by Brandt and Leong [16]. Moreover, prolonged use of these drugs is believed to be associated with severe withdrawal symptoms and a risk of dependence. Inappropriate use of these drugs is one of the most prevalent and consequential forms of prescription drug misuse, reaching values of 7.7% in such countries as the United Kingdom or North America [17].

Organizations such as the United Nations’ International Narcotics Control Board warn that the global misuse of prescription drugs such as analgesics, opioid substitution drugs, sedatives and hypnotics may soon surpass the use of illicit substances. This prediction is already becoming a reality in countries like the United States; similarly concerning data have been reported in Europe [18]. For this reason, the chronic and widespread use of Z-drugs has become a public health concern, prompting multiple campaigns aimed at reducing both their prescription and consumption [19].

The widespread use of psychoactive pharmaceuticals has raised concerns about the presence of their residues in the environment [20]. Unfortunately, while numerous studies have examined the removal of antidepressants and their effects on aquatic environments, only a small number have focused on Z-drugs and evaluated their potential risks to aquatic ecosystems [20,21]. Z-drugs enter the environment primarily through excretion in their unchanged form or as active metabolites, incomplete degradation during wastewater treatment [22] and improper disposal of unused medicines. Once released into surface waters, Z-drugs and their metabolites undergo various physicochemical and biological processes such as biodegradation, sorption, hydrolysis, and photolysis, which influence their environmental persistence and potential toxicity [23,24]. The continuous input of these compounds into aquatic ecosystems, even at trace concentrations (e.g., ng L^−1^), results in their so-called pseudo-persistence, highlighting the need for more effective removal technologies and comprehensive ecological risk assessments [21,25]. Even at low environmental concentrations, these compounds may interfere with endocrine systems and disrupt hormonal balance [26]. Consequently, their presence in aquatic environments may pose risks to non-target species, with prolonged exposure potentially resulting in chronic ecological and evolutionary effects [21]. However, available data on analytical techniques for detecting Z-drugs and their major metabolites and transformation products in environmental samples remain scarce. Data on their removal efficiency in wastewater treatment plants, their environmental fate, occurrence in various matrices, and associated ecotoxicological impacts are also limited.

This review aims to consolidate current knowledge on these aspects. To the best of our knowledge, no review of this scope has previously been published.

## 2. Scope and Methodology of the Review

**Figure 1:**
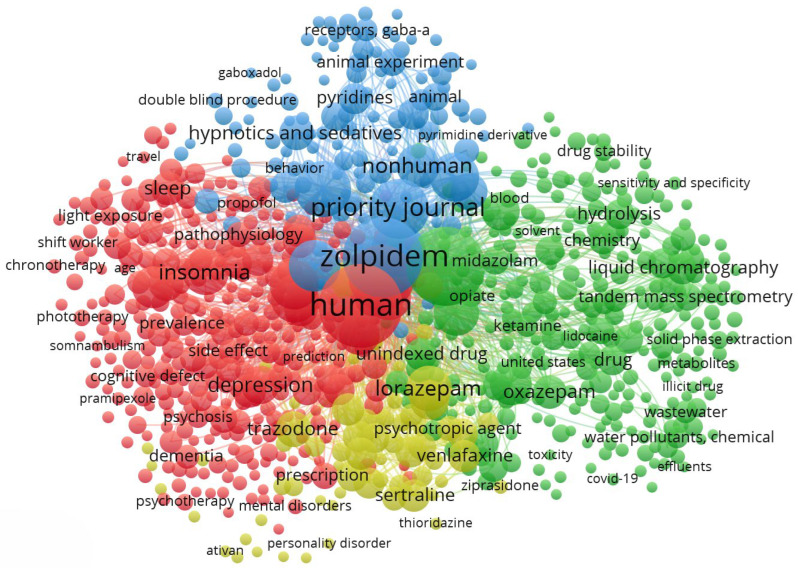
VOSviewer cluster visualization of keyword co-occurrence in Scopus-indexed publications on Z-drugs (minimum keyword occurrence = 5). Each color represents a distinct thematic cluster of related keywords.

Data for this bibliometric analysis were retrieved from the interdisciplinary abstract and citation database Scopus. The search query applied was: (“Z-drugs” OR “non-benzodiazepine” OR “zolpidem” OR “zaleplon” OR “zopiclone” OR “eszopiclone”) AND (“environment” OR “wastewater” OR “wastewater treatment” OR “WWTP” OR “effluent” OR “biodegradation” OR “sorption” OR “hydrolysis” OR “photolysis” OR “removal” OR “ecotoxicity” OR “ecotoxicological” OR “ecotoxicological effects” OR “ecotoxicity assessment” OR “persistence” OR “environmental fate”). This search yielded 418 publications, which were subsequently analyzed using VOSviewer (version 1.6.20) to construct keyword co-occurrence maps (Figure 1).

The cluster map (Figure 1) reveals three main thematic domains in Z-drug research. The first cluster is Clinical Psychiatry and Therapeutics (red cluster). This cluster centers on keywords related to insomnia and psychiatric disorders. High-frequency terms include “insomnia”, “depression”, “sleep disorders”, and names of co-prescribed psychiatric medications (e.g., “sertraline”, “venlafaxine”, “trazodone”, which are antidepressants). The co-occurrence of these terms indicates a large body of clinical research on using Z-drugs to manage insomnia in patients with mood and anxiety disorders or other comorbid conditions.

The second cluster, Pharmacological Mechanisms and Sedative Effects (blue cluster), is dominated by pharmacological and experimental terms. Notably, “GABA_A receptor”, “receptors”, “hypnotics and sedatives”, “pharmacodynamics”, “tolerance”, “dose–response”, and “animal experiment” appear prominently. This indicates a research theme focused on mechanisms of action and comparative pharmacology of Z-drugs. It reflects how Z-drugs, introduced in the 1990s as safer alternatives to older hypnotics, were extensively studied to characterize their action on the central nervous system.

A third major cluster, Environmental Fate, Ecotoxicology, and Analytical Chemistry (green cluster), is characterized by environmental science and analytical technique keywords. Terms such as “wastewater”, “water pollutants”, “effluent”, “surface water”, “biodegradation”, “ecotoxicity”, and “environmental monitoring” are clustered together, indicating a research theme on the occurrence and impact of Z-drugs in the environment. Accompanying these are numerous analytical chemistry keywords and related terms. This suggests that a growing segment of the literature deals with detecting and quantifying Z-drug residues in environmental samples (like wastewater/sewage and rivers) and assessing their persistence and ecological effects. The inclusion of terms like “COVID-19”, “pandemic”, and “wastewater-based epidemiology (WBE)” in this cluster further underscores a recent investigative angle, including the use of sewage analysis to monitor community drug use trends. For example, one study during the COVID-19 pandemic measured zolpidem and related sedatives in wastewater to estimate population-level consumption, marking the first detection of zolpidem in North American wastewater [27].

Overall, this cluster represents an environmental and analytical chemistry perspective on Z-drugs, reflecting multidisciplinary efforts to trace the “downstream” consequences of widespread Z-drug use, from advanced analytical method development to understanding environmental contamination and risks.

**Figure 2:**
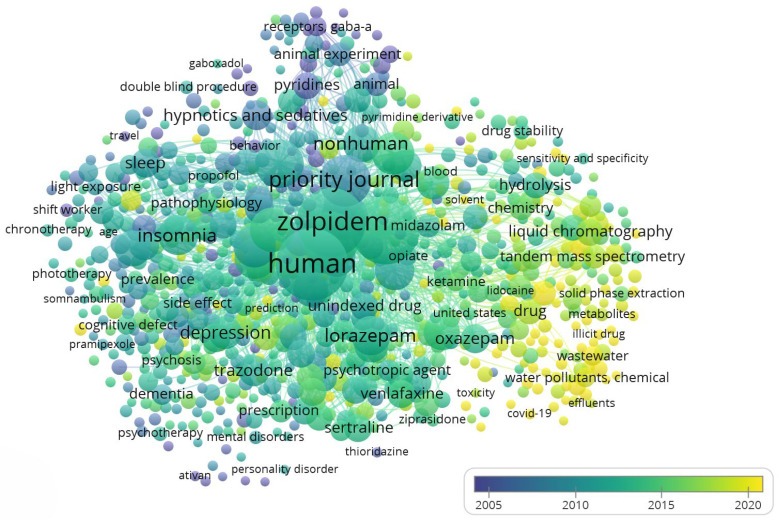
VOSviewer overlay visualization of the same co-occurrence network, with node colors indicating the average publication year of documents associated with each keyword (from ~2005 in blue to ~2020 in yellow). Warmer colors denote topics that have been studied more recently.

The overlay map (Figure 2) illustrates the temporal evolution of Z-drug research between 2005 and 2020. Earlier studies (blue–green colors) focused on clinical efficacy and pharmacology, with keywords such as insomnia, depression, and GABA_A receptor reflecting the role of Z-drugs in psychiatry and their pharmacodynamic characterization. In contrast, more recent research (yellow hues) emphasizes environmental and analytical themes, including wastewater, biodegradation, LC–MS/MS, and ecotoxicity. The appearance of COVID-19 and wastewater-based epidemiology marks the most recent expansion of this field, highlighting novel applications of sewage analysis to track population-level drug use. Intermediate terms such as sleep quality and adverse effects suggest sustained interest in clinical safety into the 2010s.

The overlay timeline results point to a chronological shift in research priorities that carries interpretative significance. Early in the analyzed period (mid-2000s), Z-drug research was primarily concerned with clinical effectiveness and drug characteristics, befitting the timeframe when Z-drugs were gaining popularity as insomnia treatments. As Z-drugs became mainstream prescriptions (global use of Z-drugs was rising annually during the 2000s–2010s), researchers addressed practical questions like how these drugs compare to older benzodiazepines, how to integrate them into the treatment of depression or anxiety, and how to mitigate their adverse effects [28,29,30]. Meanwhile, the surge of newer terms related to environmental monitoring in the late 2010s illustrates how the scientific community eventually broadened its scope. This likely corresponds to growing awareness of pharmaceutical environmental contamination as a public health issue around that time [31]. Essentially, the cluster map’s juxtaposition of psychiatric terms with environmental terms highlights an emerging “one health” perspective, integrating human health and environmental health [27,32,33].

Overall, the cluster visualization (Figure 1) and overlay (Figure 2) together illustrate a coherent narrative in the scientific literature on Z-drugs: initial research asked “How do we use these drugs safely and how do they work?” and subsequent research asks “What are the broader consequences of using these drugs at population scale?”. The thematic clusters of pharmacotherapy, pharmacodynamics, and environmental analysis each contribute a piece of this larger picture. By interpreting the maps, we see that Z-drugs are not only a topic of pharmacological interest but also a lens through which multiple disciplines intersect, from psychiatry and neuroscience to environmental chemistry and public health. This comprehensive, map-guided analysis highlights the importance of interdisciplinary awareness. Essentially, the cluster map’s juxtaposition of psychiatric terms with environmental terms highlights an emerging “one health” perspective, integrating human health and environmental health.

The impact of Z-drugs on environmental health is closely linked to their chemical structures, physicochemical and pharmacokinetic properties, as well as consumption levels at global, regional, and national scales. Therefore, these data are presented at the beginning of this section.

## 3. Physicochemical and Pharmacokinetic Properties of Z-Drugs

**Figure 3:**
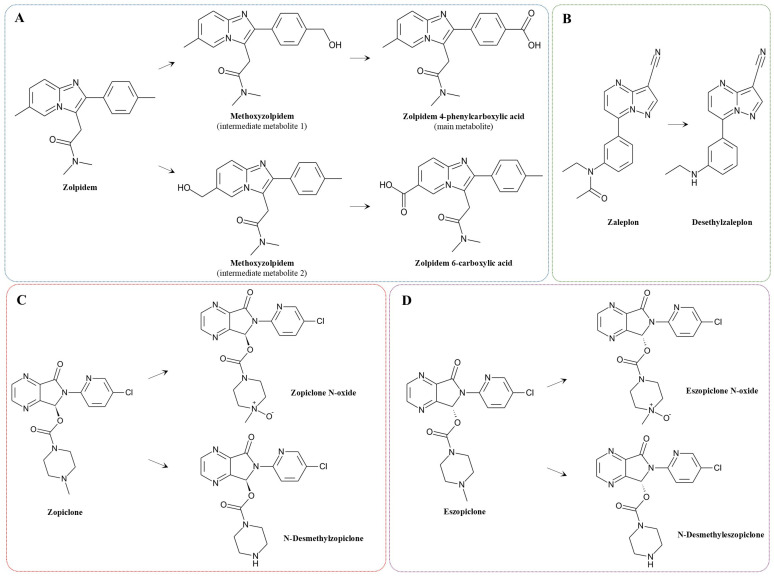
Metabolic pathways of the main Z-drugs: zolpidem (**A**), zaleplon (**B**), zopiclone (**C**), and eszopiclone (**D**) [36,37,38,39].

The structures and physicochemical properties of the main Z-drug representatives are summarized in Table 1, while their pharmacokinetic properties are presented in Table 2. The metabolic pathways of these compounds are illustrated in Figure 3.

Based on the data presented in Table 1 and Table 2, both the parent compounds and their main metabolites can enter the environment. These compounds exhibit notable differences in their physicochemical properties (e.g., lipophilicity, pKa, and water solubility, Table 1), which are likely to influence their behavior, persistence, and removal efficiencies in wastewater treatment systems. Importantly, zopiclone is a chiral hypnotic and sedative marketed as a racemate that includes (R)-zopiclone and (S)-zopiclone (named eszopiclone), exhibiting stereoselectivity in both pharmacokinetics and pharmacodynamics. The second enantiomer shows an affinity for the benzodiazepine receptor 50 times higher than the (R)-enantiomer [34]. Consequently, eszopiclone was developed as a distinct single-enantiomer drug to match the racemic drug’s therapeutic effect while reducing adverse effects [35].

Knowledge of the physicochemical and pharmacokinetic properties of Z-drugs is essential for further discussion of their environmental fate and potential ecotoxicological effects. The data summarized in Table 1 and Table 2 will be referred to in the sections discussing removal efficiency in wastewater treatment plants (WWTPs), environmental fate in aquatic systems, and possible ecotoxicological impacts, highlighting the role of both parent compounds and their metabolites.

**Table 1:** Structures and physicochemical properties of the main Z-drugs (zolpidem, zaleplon, zopiclone, and eszopiclone).

Parameter	Zolpidem	Zaleplon	Zopiclone	Eszopiclone	Ref.
Molecular formula	C_19_H_21_N_3_O	C_17_H_15_N_5_O	C_17_H_17_ClN_6_O_3_	C_17_H_17_ClN_6_O_3_active S-enantiomer of zopiclone	[36,37,38,39]
Chemical structure	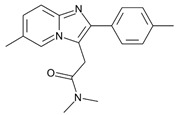	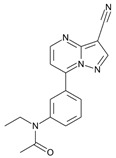	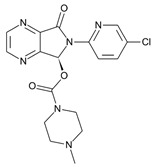	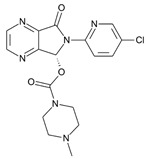	[36,37,38,39]
Molecular weight [g/mol]	307.4	305.3	388.8	388.8	[36,37,38,39]
Log P (lipophilicity)	3.02	0.9 ~1.23 pH 1–7 [40]	0.8	0.81	[36,37,38,39]
Water solubility	23 mg mL^−1^ at 20 °C	0.0403 mg mL^−1^ (predicted)	0.151 mg mL^−1^ at 25 °C	0.885 mg mL^−1^ (predicted)	[36,37,38,39]
pKa	(Strongest Basic) 5.39 (predicted)	0.28 (predicted)	8.04 (predicted)	9.2	[36,37,38,39]

**Table 2:** Pharmacological properties of Z-drugs (zolpidem, zaleplon, zopiclone, and eszopiclone).

Parameter	Zolpidem	Zaleplon	Zopiclone	Eszopiclone	Ref.
Dose range	5–10 mg * 6.25–12.5 mg **	5–20 mg	3.75–7.5 mg	1–3 mg	[30]
Absorption Tmax	1.6 h [35] 1–2.5 [28]	Rapidly and almost completely absorbed following oral administration [41] 0.7–1.4 h [28]	Rapidly absorbed following oral administration <15 min [36] 1.5–2 h [28]	1 h [39] 1–1.5 h [30]	[30,36,37,38,39]
Protein binding	92.5 ± 0.1%	Approximately 60%	Approximately 45%	52–59%	[36,37,38,39]
Oral bioavailability	65–70%	30%	75–80%	75–80%	[30]
GABA_A receptor selectivity	α1-preferring	α1-preferring	Nonselective	Moderately selective (α1/α2)	[36,37,38,39,42,43,44]
Major metabolism	CYP3A4 (major), CYP1A2 and CYP2C9	Aldehyde oxidase (major), CYP3A4	CYP3A4, CYP2C8	CYP3A4, CYP2E1, CYP2C9	[30,36,37,38,39,42,43,44]
Route of elimination	Urine (~56%, metabolized) Less than 1% in the urine as the parent drug	Urine (71%), feces (17%) Less than 1% in the urine as the parent drug	Urine (~75%, metabolites)	Urine (~75%, metabolites) 10% in the urine as the parent drug	[36,37,38,39]
Elimination half-life	2.6 and 2.5 h, for the 5 and 10 mg tablets [35] 1–2.5 [28]	1 h [28,41]	5–6 h [28,36]	6.1 h [39] 6–7 h [30]	[30,36,37,38,39]

* Zolpidem IR—IR immediate-release preparation. ** Zolpidem ER—ER extended/controlled-release preparation.

## 4. Consumption of Z-Drugs

**Figure 4:**
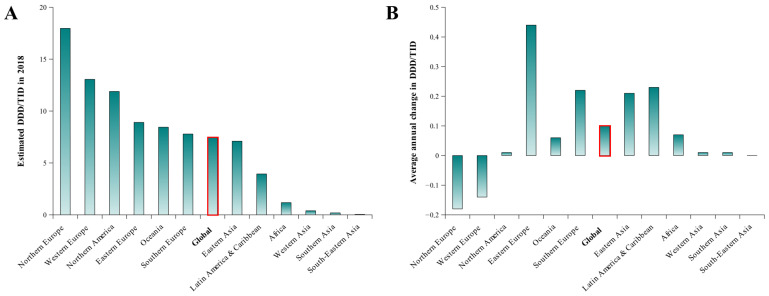
Estimated DDD/TID values for Z-drugs in 2018 (**A**) and average annual change in DDD/TID values (**B**) for Z-drugs across regions [41].

Ma et al. [41] reported global patterns of Z-drug use across 67 countries and regions between 2008 and 2018. Their analysis was based on worldwide pharmaceutical sales data obtained from the IQVIA Multinational Integrated Data Analysis System for the same period representing approximately 75% of the global population. Z-drug consumption was quantified as defined daily doses per 1000 inhabitants per day (DDD/TID). Trends at the global, regional, and country levels were assessed using linear mixed-effects models. Countries were also stratified according to income category for supplementary analyses, and univariable linear regression models were applied to examine relationships between Z-drug use, gross domestic product (GDP), and the prevalence of selected medical conditions [41]. Between 2008 and 2018, overall global use of Z-drugs rose by 3.28%, reaching an estimated DDD/TID value of 7.48 in 2018, and as one of the top 300 most widely produced pharmaceuticals, zolpidem remained the most widely sold Z-drug. Consumption patterns for zolpidem showed no statistically significant change over time, a finding that was also observed for zaleplon and zopiclone, while eszopiclone use exhibited a clear upward trend (average annual change in DDD/TID was 0.006). In 2018, the highest levels of Z-drug consumption were reported in Sweden (30.77), Norway (30.33), Luxembourg (21.90), Ireland (21.86), Belgium (20.78), Estonia (20.75), and the Czech Republic (19.83), whereas the lowest levels were seen in the Philippines (0.04), Thailand (0.03), Saudi Arabia (0.03), Turkey (0.02), French West Africa (0.02) and Kuwait (0.01) [41]. Z-drug consumption was high in North Europe, West Europe, North America and Japan, with values of 17.97, 13.06, 11.89, and 15.53 DDD/TID, but was very low in Africa and Asia, with DDD/TID lower than 10. Overall use was substantially greater in high-income countries than in middle-income countries; however, during the study period, consumption declined in high-income settings while increasing in middle-income ones. Z-drug consumption rates were found to be associated with GDP as well as the prevalence of mental and neurological disorders, chronic respiratory diseases, cardiovascular diseases, and cancer [41]. These global consumption patterns and regional trends are illustrated in Figure 4.

These data are in agreement with the national trends in the dispensing of reimbursed hypnotics observed in European countries—Finland, Norway, Denmark, Sweden, Greece, France, the Netherlands, Spain and the United Kingdom—where it has become a public health issue and has led to multiple campaigns to reduce both the prescription and consumption of BZD/Z-drugs [8,12,19]. For example, in France between 2012 and 2022, a nearly 40% decrease in Z-drug dispensing was observed, driven primarily by a 69.15% drop in zolpidem dispensing [8]. On the other hand, a statistically significant linear increase in zolpidem use was observed in the Republic of Serbia from 2006 to 2021 [45]. The COVID-19 global pandemic also played a role in globally increasing the diversion of prescribed Z-drugs [13,46,47]. A need to improve the quality of consumption and reduce the negative impact of Z-drugs has been observed in Argentina [48] and Japan [49].

As was mentioned, the misuse of Z-drugs outside medical supervision is a well-recognized issue and constitutes a growing public health concern [4,13,17,18,30]. The diversion of prescribed Z-drugs has also been linked to drug-facilitated sexual assaults (DFSAs), including date-rape cases, where central nervous system depressants are administered forcibly or surreptitiously to impair victims’ behavior, induce vulnerability or loss of consciousness, and frequently cause anterograde amnesia. Severe health outcomes, including fatal overdoses, must be taken into account not only among high-risk populations such as opioid users, but also within the general population. It is difficult to quantify the scale of non-medical use of Z-drugs, largely because many countries lack systematic surveillance and data-collection mechanisms. Therefore, enhanced awareness, monitoring, and response initiatives should be strengthened at both national and international levels to address emerging patterns of prescription drug diversion [4,13,17,30].

The widespread use of these drugs has raised concerns about the presence of their residues in the environment. The environmental risk assessment (ERA) of Z-drugs evaluates their potential impacts on aquatic and terrestrial ecosystems following human use and subsequent environmental release [50,51]. In Phase I, the potential of a drug to migrate beyond aquatic systems and bioaccumulate in the food chain is assessed, and its Predicted Environmental Concentration (PEC) in surface waters is calculated. The PEC is estimated using consumption data, excretion rates (parent compounds and metabolites), removal efficiency in WWTPs, and dilution factors in receiving waters. If the PEC is <0.01 μg/L and no other environmental concerns are identified, the product is considered unlikely to pose a risk to the environment under prescribed use. If the PEC is ≥0.01 μg/L, a Phase II assessment addressing environmental fate and effects is required. Even at low environmental release levels, highly lipophilic compounds and potential endocrine-disrupting substances may warrant evaluation in Phase II. Based on toxicity studies, a Predicted No-Effect Concentration (PNEC) is then derived [50,51]. Environmental risk is typically expressed as a Risk Quotient (RQ):

RQ = PEC/PNEC

RQ < 1 → Low environmental risk

RQ ≥ 1 → Potential environmental concern

Currently, the pharmaceutical risk assessment framework proposed by the European Medicines Agency (EMA) is based on standardized protocols developed by the Organization for Economic Co-operation and Development (OECD) [50,51].

Z-drugs are often only partially removed by conventional wastewater treatment processes (Section 6). In the environment, they may undergo various transformation processes that affect their persistence and overall fate (Section 7), leading to measurable concentrations in wastewater effluents and receiving surface waters (Section 8). Due to their psychoactive properties and mechanism of action on γ-aminobutyric acid (GABA) receptors, particular concern has been raised about their potential to induce behavioral and neurological effects in non-target aquatic organisms (Section 9). However, all such investigations require the availability of reliable analytical methods for the determination of Z-drugs in environmental matrices.

## 5. Analytical Methods for Determining Z-Drugs in the Environmental Matrices

Due to their widespread use in the treatment of sleep disorders and their pharmacological activity (Table 2), monitoring of these compounds is extremely important from an environmental protection perspective. Due to the very low concentrations of these compounds detected in environmental samples (in the ng L^−1^ range) and the complex nature of the matrices analyzed, their determination requires the use of advanced analytical techniques characterized by high sensitivity and selectivity. In this section, the most commonly used analytical methods for analysis of Z-drugs in environmental samples will be discussed.

### 5.1. Analytical Techniques Used in the Determination of Z-Drugs

Monitoring of Z-drugs in the environment is a relatively new area of research, and the number of available literature reports is still limited. So far, analyses of these compounds have focused mainly on samples of wastewater, surface water, bottom sediments, and fish tissues (Table 3). The dominant analytical technique remains liquid chromatography coupled with tandem mass spectrometry (LC-MS/MS, UHPLC-MS/MS). In the majority of cases, a targeted approach was used, focusing on the determination of selected substances, without a screening strategy. In such cases, the dominant mass analyzers were triple quadrupole (QqQ) [23,25,26,33,52,53,54,55] and hybrid quadrupole-ion trap (QTRAP) systems [56,57], as well as MRM mode (Multiple Reaction Monitoring), which ensures high selectivity and sensitivity. In contrast, in untargeted analyses aimed at detecting as many compounds as possible (including metabolites and environmental transformation products), high-resolution mass analyzers such as hybrid quadrupole-time-of-flight (QTOF) [58] and quadrupole-orbitrap (Q-Orbitrap) [59] systems were used. In some cases, supercritical fluid chromatography (SFC-MS/MS) [54] and fluorescence spectroscopy [60] were also applied as alternative techniques for determining selected Z-drugs. The most commonly used ion source was electrospray ionization (ESI) [23,25,26,33,52,53,55,56,57,58,61], while in some cases heated ESI (HESI) [59,62] was applied, which increases ionization efficiency in the presence of complex matrices.

In the analysis of Z-drugs using the techniques presented above, various types of chromatographic columns have been employed (Table 3). Most commonly, stationary phases containing octadecyl groups (C18) were used [23,25,26,57,58,59,61]. In some cases, phenyl-based [33] and fluorinated phases [52,56] were applied. For the enantiomer analysis of zopiclone (E1- and E2-zopiclone; R/S-enantiomers), a chiral column [53] was applied, where cellobiohydrolase serves as the chiral selector. Elution was typically performed in gradient mode, using mobile phases composed of water (with the addition of formic or acetic acid) and methanol or acetonitrile, which allows separation of compounds differing in polarity [23,25,26,33,52,55,56,57,58,59,61]. However, when a chiral column was used, isocratic elution was applied [53]. 

Brieudes and co-workers [23] investigated various parameters affecting chromatographic separation of 68 drugs, including Z-drugs and their metabolites. They examined factors such as stationary phase type (Acquity^®^ UPLC BEH C18, Acquity^®^ UPLC BEH Shield RP18, and Acquity^®^ UPLC HSS T3, all manufactured by Waters, Milford, MA, USA), flow rate (0.3–0.6 mL/min), column temperature (30–60 °C), mobile phase composition (water–methanol and water–acetonitrile systems), and the influence of acid addition (formic and acetic acid at 0.05–1% *v*/*v*), as well as the presence of salts (ammonium formate and ammonium acetate in the range of 1–10 mM). The goal was to achieve the best compromise between peak shape, resolution, and signal-to-noise ratio. Ultimately, the most favorable conditions were provided by an Acquity^®^ UPLC BEH C18 column (1.7 μm; 2.1 mm × 100 mm, Waters, Milford, MA, USA) heated to 40 °C. A gradient using water and acetonitrile, both with the addition of 0.1% (*v*/*v*) acetic acid, was applied at a flow rate of 0.6 mL/min.

A detailed list of analytical methods used for determining Z-drugs and their metabolites in environmental samples is provided in Table 3.

### 5.2. Sample Pretreatment Strategies

Sample preparation is a critical stage in the analytical workflow, especially when dealing with complex environmental matrices. The choice of an appropriate sample preparation method depends on several factors, including the type of matrix, the physicochemical properties of the target compounds (Table 1), and the requirements of the analytical technique used. In the analysis of Z-drugs in environmental samples, the most commonly applied method was solid-phase extraction (SPE), which provides both removal of matrix interferences and preconcentration of analytes, an essential requirement given the very low concentrations typically observed for these compounds. Various SPE formats were employed, including conventional cartridge-based systems [23,33,53,54,55,56,59,61,62], extraction discs [25], and automated on-line SPE setups [57] directly coupled to the analytical instrumentation (usually preceded by filtration and pH adjustment).

Racamonde and colleagues [55] evaluated the suitability of different solid-phase extraction (SPE) cartridges for the preparation of wastewater samples. Three types of sorbents were compared: Oasis HLB (Hydrophilic–Lipophilic Balance), Oasis MAX (Mixed-Mode Anion eXchange), and Oasis MCX (Mixed-Mode Cation eXchange). Oasis HLB contains both hydrophilic and lipophilic fragments, making it a versatile option for a wide range of analytes. Oasis MAX is a polymeric sorbent with basic functional groups enabling anion exchange, whereas Oasis MCX incorporates acidic groups allowing cation exchange. The authors investigated parameters such as sample pH, breakthrough volume, and final extract volume to assess analyte recovery and matrix effects. Ultimately, the best results were obtained with Oasis MCX cartridges, which provided higher recoveries of basic compounds and allowed the use of a simplified elution procedure with methanol containing ammonia. Using this protocol for zolpidem, recoveries were approximately 100% with a relative standard deviation (RSD) of 5% and a limit of quantification (LOQ) of 1.0 ng L^−1^ for influent samples, while for effluent samples recoveries reached about 110% with an RSD of 3% and an LOQ of 0.9 ng L^−1^ [55].

For biological samples such as fish tissues, acetonitrile-assisted extraction was used in combination with homogenization using zirconium beads and subsequent centrifugation [62]. In their study on the untargeted analysis of polar contaminants in freshwater sediment samples, Terzic and co-workers [58] applied Soxhlet extraction followed by fractionation of the extract on a silica gel column. On the other hand, Ting and co-workers [52], in their study on the monitoring of 68 abused drugs, used dispersive liquid–liquid microextraction (DLLME), a rapid and efficient analyte enrichment technique with minimal solvent consumption. In some cases, sample preparation was limited to simple centrifugation [26].

The applied sample preparation protocols for isolation and enrichment of Z-drugs from environmental matrices are presented in Table 3.

### 5.3. Advantages and Limitations of Analytical Techniques

Targeted LC-MS/MS methods, especially those based on triple quadrupole (QqQ) or QTRAP systems operated in MRM mode, remain the primary analytical tools for the determination of Z-drugs in environmental samples. Their main advantages are high sensitivity and selectivity, which allow the detection of these compounds at very low concentrations (ng L^−1^) even in complex matrices such as wastewater. When optimized, these methods can be used to quantify both parent compounds and selected metabolites or transformation products [23,25,26,33,52,53,54,55,56,57,61]. High-resolution mass spectrometry (QTOF, Orbitrap systems), typically applied in suspect and non-target screening, enables the detection of a wider range of compounds, especially for metabolites lacking reference standards [58,59]. However, HRMS methods often exhibit slightly higher detection limits and less precise quantification compared to targeted MRM strategies. Therefore, both approaches are complementary rather than interchangeable.

In studies where enantiomeric resolution is important, for example in wastewater-based epidemiology (WBE) or when considering the potential biological differences between enantiomers, chiral chromatographic methods are required. Castrignanò et al. demonstrated that the use of a CBH chiral column enabled effective separation of zopiclone enantiomers while maintaining high recoveries, very low method detection limits (sub-ng L^−1^ concentration range), and good precision [53]. This type of analysis is particularly valuable, as enantiomers can differ in biological activity, environmental fate, and transformation pathways.

Matrix effects constitute one of the major limitations of LC–MS/MS analysis of Z-drugs, particularly in wastewater samples. Depending on the matrix and compound, both ion suppression and enhancement have been reported. Brieudes et al. observed a +30% enhancement of the zolpidem signal in river water [23], whereas Yuan et al. documented strong suppression for zaleplon in wastewater influent (−23%) and effluent (−13%) in psychiatric hospital WWTPs, and milder effects in municipal WWTPs (from −9% to +9%) [25]. Such variability underscores the need for isotopically labelled internal standards, as they effectively correct for matrix-induced fluctuations in ionization efficiency [23,26]. Beyond internal standardization, ionization conditions themselves play an important role in method performance. Electrospray ionization (ESI) was the most commonly used ion source across the reviewed studies, and recent work by Brieudes and co-workers demonstrated that the addition of EDTA to samples increased the signal intensities of several pharmaceuticals, including zolpidem, by chelating residual metal ions that would otherwise form metal–analyte adducts and/or suppress analyte ionization in the source [23]. Such simple modifications can therefore contribute to improved sensitivity when compatible with the analytical procedure.

In addition to matrix-related signal variability, method performance in environmental analyses of Z-drugs is often limited by insufficient analytical sensitivity relative to the low concentrations present in surface waters and wastewater. Reported LOQs vary considerably between target compounds and across laboratories, which directly affects detection frequency. For instance, Brieudes et al. achieved LOQs as low as 0.1–0.2 ng L^−1^ for zolpidem and its metabolites (zolpidem 4-phenylcaboxylic acid and zolpidem 6-carboxylic acid), enabling reliable quantification in river water at concentrations up to several ng L^−1^. In contrast, zopiclone exhibited higher LOQs (0.9 ng L^−1^), and its N-oxide metabolite (LOQ 0.8 ng L^−1^) remained undetected in all analyzed river samples [23]. Yuan et al. reported LOQ values of 20 ng L^−1^ (effluent) and 40 ng L^−1^ (influent), which exceeded the concentrations found in several wastewater samples (≤33 ng L^−1^). As a result, samples frequently remained below the method’s limit of quantification [25]. This highlights the necessity of optimizing extraction and concentration strategies when analytes occur near or below the ng L^−1^ range.

The determination of metabolites introduces further analytical challenges. Metabolites frequently show lower recoveries and poorer stability than parent compounds, making their detection more difficult. In the study by Brieudes et al., zopiclone recovery was 76%, while its N-oxide metabolite reached only 47%, contributing to its non-detection in environmental samples [23]. Similar limitations were reported by Castrignanò et al., where relatively high LOQ values (312.5–320.8 ng L^−1^) prevented the detection of zopiclone enantiomers in wastewater despite adequate recoveries (80–83%) [53]. Another important consideration is that metabolites may occur at higher concentrations than the parent compound. Brieudes et al. observed that zolpidem 4-phenylcaboxylic acid and zolpidem 6-carboxylic acid reached 3.75 ng L^−1^ and 0.63 ng L^−1^ in river water, respectively, compared to only 0.18 ng L^−1^ for zolpidem [23]. Therefore, omitting metabolites from environmental monitoring could lead to a substantial underestimation of total contaminant loads.

Overall, the analytical determination of Z-drugs in environmental samples relies on targeted LC–MS/MS as the most sensitive and selective approach, complemented by HRMS for broader screening and metabolite detection. Method performance is strongly affected by ionization conditions, matrix effects, and the very low environmental concentrations of both parent compounds and metabolites. Metabolites remain particularly challenging due to lower recoveries, despite their frequent environmental relevance. These observations, together with the considerable variability in LOQs and recoveries reported across studies, demonstrate that no single method is universally applicable under all environmental conditions. Continued optimization and the use of isotopically labelled internal standards are essential, while the current lack of standardized protocols highlights the need for method harmonization to ensure comparable and reliable monitoring of Z-drugs in environmental matrices.

## 6. Efficiency of Removal of Z-Drugs in WWTPs

The physicochemical properties presented in Table 1, including lipophilicity (log P), water solubility, and pK_a_, might play a critical role in influencing the removal efficiency of Z-drugs in conventional WWTPs.

Log P, a measure of a compound’s lipophilicity, is commonly used to predict the tendency of pharmaceuticals to sorb onto solid particles, such as activated sludge or suspended organic matter. Although zolpidem is more lipophilic (log P = 3.02), the other compounds exhibit log P values below 3. According to the literature, such values are associated with limited sorption onto activated sludge and, consequently, potentially lower removal efficiency via conventional sorption-based treatment processes [63]; in turn, their water solubility further influences retention in the aqueous phase and may limit the effectiveness of sorption and bioadsorption in activated sludge systems. Compounds such as zolpidem and eszopiclone, which exhibit higher water solubility than zaleplon and zopiclone (Table 1), might therefore be removed less efficiently through biological and physicochemical treatment in conventional WWTPs [64]. Additionally, pK_a_ determines the degree of ionization under the typical pH range of wastewater (6.5–8.5). In the Z-drugs analyzed, pK_a_ values range from very low (zaleplon, 0.28) to higher values (zopiclone/eszopiclone, 8.04/9.2), with zolpidem at approximately 5.39 (Table 1). Ionization may influence sorption, as non-ionized molecules tend to adsorb more readily onto sludge, whereas ionized forms remain in the aqueous phase [63]. In practice, some Z-drugs may exist in wastewater as partially or fully non-ionized species, which does not necessarily result in effective sorption or sedimentation, but could affect bioavailability and susceptibility to biological degradation.

Taken together, these observations indicate that variability in the removal efficiency of Z-drugs in WWTPs might be associated, among other properties, with lipophilicity, water solubility, and pK_a_.

To achieve a comprehensive understanding of Z-drug removal in wastewater treatment plants, it is essential to quantify the proportion of the drug excreted as the parent compound versus its metabolites (Table 2). For instance, zolpidem is largely excreted as metabolites, with less than 1% appearing unchanged in urine. This low concentration in wastewater is consistent with findings from various locations in Spain, where influent wastewater contained zolpidem at levels ranging from 2.9 to 7 ng L^−1^ [57]. Importantly, the pharmacokinetic characteristics summarized in Table 2, including extensive metabolism, short elimination half-lives, and dominant urinary excretion of metabolites, suggest that wastewater treatment plants are exposed predominantly to transformation products rather than parent Z-drugs. Moreover, the oral bioavailability and administered dose range (Table 2) directly influence the quantity of parent compounds and metabolites entering wastewater. Drugs with high oral bioavailability, such as zolpidem, zopiclone, and eszopiclone (~65–80%), are extensively metabolized in humans, resulting in a predominance of metabolites rather than the parent form in sewage. As only a small percentage of the administered dose is excreted unchanged for most Z-drugs, influent concentrations of parent compounds may underestimate the actual pharmaceutical load entering WWTPs. Additionally, the presence of conjugated or labile metabolites may contribute to apparent variability in reported removal efficiencies, as these compounds can undergo deconjugation or transformation back to parent forms during wastewater treatment. Consequently, removal efficiencies calculated solely on the basis of parent compound concentrations should be interpreted with caution, as they may not fully reflect the fate of the total Z-drug-related chemical burden within WWTPs. Additionally, high plasma protein binding, as observed for zolpidem (~92.5%; Table 2), may limit the free fraction in vivo but can undergo partial desorption under wastewater conditions, increasing the availability of both parent compounds and metabolites for biological and physicochemical removal processes [65]. Coupled with short elimination half-lives and rapid absorption, this continuous input contributes to a pseudo-persistent presence of Z-drugs and their metabolites in WWTPs [66]. However, experimental data on these processes and their quantitative impact on removal efficiencies remain scarce.

Municipal wastewater treatment plants are primarily designed to remove conventional organic and nutrient pollutants such as carbonaceous, nitrogenous, and phosphorus compounds [26]. The most common biological process employed in these facilities is the Conventional Activated Sludge (CAS) system, which has been used since 1914 for municipal wastewater treatment [67,68]. This process relies on aerobic biological oxidation, where microorganisms decompose organic matter in the presence of oxygen, producing carbon dioxide and nitrogen gases as final products [68]. While the CAS process is effective in removing typical organic contaminants, it faces several operational challenges, including sludge bulking, low biomass concentration, poor flocculation, and reduced organic matter removal efficiency, which are further aggravated by increasing influent flow [69,70]. However, the most significant limitation of modern WWTPs is that they are not specifically designed to eliminate emerging contaminants, such as pharmaceuticals, including Z-drugs [26]. Conventional biological treatment systems are not optimized to degrade persistent and structurally complex pharmaceutical compounds, which often pass through the treatment process either unchanged or as biologically active metabolites [26,55].

This limitation is exemplified by studies on zolpidem. In Czech WWTPs employing conventional biological treatment methods, the removal efficiency of zolpidem averaged 47%, with the compound detected in 9 out of 10 facilities—ranging from 1 ng L^−1^ to 55 ng L^−1^ in influent and from 1 ng L^−1^ to 19 ng L^−1^ in effluent [26]. In another study, a WWTP utilizing a combination of primary and secondary activated sludge treatment was found to contain zolpidem in both influent (up to 3.9 ng L^−1^) and effluent (1.5–1.9 ng L^−1^), indicating that a substantial fraction of the compound persisted after treatment [55].

**Figure 5:**
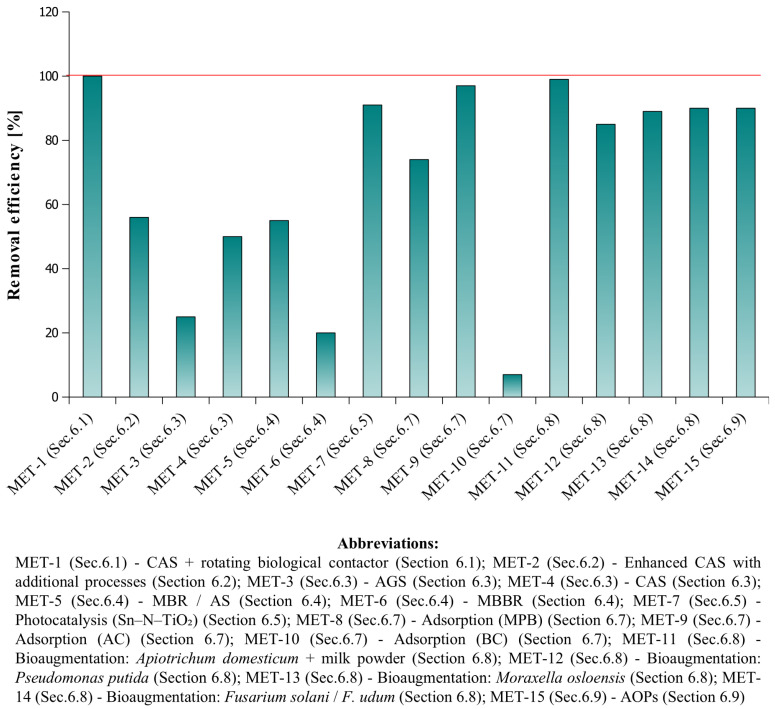
Comparison of Removal Efficiencies of Z-drugs Across Various Treatment Technologies.

These findings clearly demonstrate that conventional biological processes in WWTPs are only partially effective in removing pharmaceuticals, including Z-drugs. This highlights the urgent need for targeted and advanced treatment strategies capable of effectively mitigating pharmaceutical contamination and minimizing the release of these bioactive compounds into the aquatic environment. In response to the limited efficiency of conventional biological systems, researchers have increasingly focused on developing and evaluating innovative technologies aimed at enhancing the removal of pharmaceuticals from wastewater. Recent research has proposed and evaluated several innovative treatment approaches, including: (1) conventional activated sludge systems enhanced with rotating biological contactors, (2) various optional processes integrated into CAS systems, (3) aerobic granular sludge (AGS) technology, (4) membrane bioreactors (MBR) and moving bed biofilm reactors (MBBR), (5) photocatalytic degradation using Sn–N–TiO_2_ under sunlight, (6) exposure to light stress and other extreme conditions, (7) adsorption techniques employing magnetite–pine bark (MPB) and biochar (BC) sorbents, (8) bioaugmentation of activated sludge systems, (9) advanced oxidation processes (AOPs), and (10) ecological approaches such as algal ponds and constructed wetlands. The effectiveness of the removal of Z-drugs from wastewater using such modified or alternative technologies is described below. Table 4 provides a detailed comparison of the examined treatment technologies, including their operational characteristics and mechanisms. Figure 5 illustrates the removal efficiencies reported for each of the evaluated treatment technology.

### 6.1. Conventional Activated Sludge System Combined with Rotating Biological Contactor

In Sri Lanka, among 36 detected pharmaceutically active compounds (PACs), zolpidem was detected only twice and at low concentrations. Application of enhanced biological treatments, such as additional rotating biological contactors, significantly improved overall removal efficiency, with zolpidem achieving a 100% removal rate in the evaluated wastewater treatment plants. Its complete elimination may result from high biodegradability, efficient adsorption, or effective degradation within these enhanced systems [70].

### 6.2. Various Optional Processes Integrated into CAS Systems

In South Korea, conventional activated sludge (CAS) systems were complemented by additional processes, including coagulation/flocculation, both anoxic and aerobic biological degradation, and disinfection by chlorination or UV treatment, which collectively enhance the removal efficiency of various compounds, including pharmaceuticals such as zolpidem. In wastewater treatment plants, zolpidem was detected at low concentrations in both influent and effluent, with mean values of 3.6 ng L^−1^ and 1.4 ng L^−1^, respectively. Its main metabolite, zolpidem 4-phenylcarboxylic acid, was present at significantly higher concentrations in influent (105.7 ng L^−1^) and effluent (79.5 ng L^−1^), corresponding to a removal efficiency of only 10.1%. The overall removal efficiency of zolpidem was found to be 56.4%, ranging from 34.6% to 76.8%. This relatively high efficiency, compared to other detected compounds (excluding amphetamine, ephedrine, and methamphetamine), is likely related to its physicochemical properties, particularly a log P > 3, which favors adsorption to particulate matter and thus facilitates its removal through biological degradation. Pharmaceuticals with log P < 3 typically do not sorb to suspended solids and are less efficiently removed. These findings indicate that the application of enhanced CAS processes can significantly improve the removal of zolpidem in WWTPs, achieving higher efficiencies than for many other compounds present in the influent [24].

### 6.3. Aerobic Granular Sludge (AGS) Technology

In Sweden, the removal efficiency of zolpidem has been investigated in both aerobic granular sludge (AGS) and conventional activated sludge (CAS) systems, focusing on its transformation under varying redox conditions [67]. AGS represents a modern and energy-efficient wastewater treatment technology in which microorganisms form dense, compact granules stabilized by extracellular polymeric substances (EPS). This structure promotes high biomass concentrations, excellent settling properties, and diverse redox micro-niches that facilitate the removal of a wide range of organic micropollutants (OMPs) and antibiotics [67]. Despite these advanced characteristics, zolpidem exhibits limited biodegradability. Under oxic conditions, the biodegradation rate constants (k_bio) ranged between 0.10–0.11 L gSS^−1^ d^−1^, while under anoxic conditions, k_bio ≤ 0.04 L gSS^−1^ d^−1^, indicating negligible degradation [67]. Significant transformation occurred only in oxic environments for both AGS and CAS, with higher reaction rates (Δk_bio > 0.04 L gSS^−1^ d^−1^) compared to anoxic conditions. Similar k_bio values observed in oxic CAS and AGS systems suggest that AGS does not substantially enhance zolpidem removal. The residual fraction of zolpidem in treated effluent exceeded 50% for CAS and 75% for AGS, confirming its persistence during biological treatment. Therefore, zolpidem can be classified as moderately biodegradable, yet showing strong resistance to microbial transformation, as reflected by its low k_bio values and high residual concentrations [67]. In comparison, naproxen exhibits much higher biodegradation rates (k_bio = 2.55 L gSS^−1^ d^−1^ in oxic AGS), highlighting zolpidem’s limited susceptibility to microbial degradation. The deviations (±25%) between modeled and measured residuals of zolpidem, losartan, and trimethoprim in CAS effluents were likely caused by differences in nutrient availability and microbial community composition. Moreover, variations between full-scale WWTP measurements (conducted in November) and laboratory batch tests (performed in February) may have further contributed to these discrepancies. Collectively, these results emphasize the critical role of oxygen availability in zolpidem degradation and illustrate the persistent challenge of effectively removing Z-drugs in current biological wastewater treatment systems [67].

### 6.4. Membrane Bioreactors (MBRs) and Moving Bed Biofilm Reactors (MBBRs)

In terms of differences between AGS and CAS systems, the type of biological treatment employed in WWTPs has a substantial impact on zolpidem removal efficiency. A comparative study conducted in Slovenia demonstrated significant variability among treatment configurations. The activated sludge (AS) and membrane bioreactor (MBR) systems achieved the highest zolpidem removal efficiencies, ranging between 50–60%, whereas the moving bed biofilm reactor (MBBR) exhibited the lowest performance, with removal efficiencies around 20%. For zolpidem specifically, the average removal efficiency across all investigated WWTPs was 52%, with a wide range of 11% to 81%, indicating strong dependence on operational conditions and treatment design. Its main metabolite, zolpidem–phenyl-carboxylic acid (Figure 3), showed a similar mean removal efficiency of 52%, but with an even broader range from –2% to 74%, suggesting potential formation–removal dynamics within treatment processes. These findings emphasize that while conventional biological systems can achieve partial elimination of zolpidem and its metabolites, their persistence across treatment configurations underscores the necessity of implementing advanced quaternary treatment processes, such as ozonation or activated carbon adsorption, to enhance the removal of psychoactive pharmaceuticals resistant to biodegradation [21].

### 6.5. Photocatalytic Degradation Using Sn–N–TiO_2_ Under Sunlight

These investigations have explored the photocatalytic degradation of Z-drugs, with particular focus on extending photoactivity into the visible light region to enhance the utilization of solar energy in wastewater treatment. Using zopiclone as a model compound, studies demonstrated that up to 91% of zopiclone could be degraded within 120 min under sunlight when treated with 0.25% Sn-doped N-TiO_2_ (Sn–N–TiO_2_) photocatalysts. These photocatalysts are environmentally friendly and promote photodegradation through the synergistic effect of tin (Sn) and nitrogen (N) doping. The photocatalytic efficiency is further improved by enhanced charge carrier separation and prolonged carrier lifetime, resulting in effective pollutant removal. The degradation process of zopiclone under these conditions also leads to the formation of transformation products, such as 2-chloropyridine. These findings highlight the potential of photocatalytic approaches using Sn–N–TiO_2_ under sunlight as a promising strategy for the removal of persistent Z-drugs from wastewater [71].

### 6.6. Exposure to Light Stress and Other Extreme Conditions

Solely under natural light exposure, zolpidem in solid form for instance exhibited no significant photolytic degradation, whereas solutions of zolpidem tartrate became yellow if not protected from light, indicating its photolytic instability. To simulate more severe light stress, other experiments were conducted according to ICH guidelines, with a total illumination of 1.2 million lux hours and an integrated near-ultraviolet energy of 200 Wh/m^2^. Under these conditions, significant photodegradation occurred, confirming the sensitivity of zolpidem to intense light. In comparison, under typical wastewater treatment conditions, including neutral pH, moderate temperatures, and low light exposure, zolpidem remained largely stable, and no substantial degradation was observed. Significant degradation was only achieved under extreme conditions, such as strongly alkaline solutions, elevated temperatures, or intensive photolysis, leading to the formation of transformation products including zolpacid, oxozolpidem, zolpyridine, and zolpaldehyde. Preliminary oxidative degradation experiments using hydrogen peroxide solutions at concentrations of 0.3–3% did not result in appreciable degradation. Light was found to initiate and promote oxidative breakdown of the drug; consequently, the same degradation products were obtained during both oxidation and photolysis, with photolysis producing higher amounts of these products. Additionally, zolpidem tartrate demonstrated stability toward neutral hydrolysis and thermal degradation. Unfortunately, the extreme conditions used in these experiments are difficult or practically impossible to implement in standard wastewater treatment plants without posing risks to the overall biological process and the safety of the facility. Intensive photolysis, or more specifically UV light, could potentially be applied locally, for example in quaternary treatment processes, such as ozonation combined with UV, UV photoreactors, or TiO_2_/Sn–N–TiO_2_ photocatalysis. In these cases, the drug can be effectively degraded, but such approaches require additional infrastructure and are not part of conventional biological treatment systems [72].

### 6.7. Adsorption Techniques Employing Magnetite–Pine Bark (MPB) and Biochar (BC) Sorbents

The removal efficiency of zopiclone from municipal wastewater was investigated using also various adsorption-based approaches, highlighting the influence of sorbent type. In batch adsorption tests, magnetite–pine bark (MPB) achieved 73.5% removal, demonstrating substantial efficacy, while activated carbon (AC) reached the highest removal at 96.7%. In contrast, biochar (BC) showed a considerably lower removal rate of 6.7%. In a pilot-scale study, zopiclone was treated using a continuous-flow adsorption system with a large column (bed volume: 21 L) packed with a mixture of low-cost MPB and BC. Before treatment, the average zopiclone concentration in the wastewater effluent was 0.49 µg L^−1^. After passing through the pilot-scale column, the concentration decreased significantly to a range of <0.01–0.13 µg L^−1^, corresponding to an overall removal efficiency of 73.5%. These results indicate that MPB is an effective and low-cost sorbent for zopiclone removal at both batch and pilot scales, while AC exhibits the highest removal in batch experiments, and BC alone is less effective. Regeneration processes were also explored; however, they revealed challenges such as iron leaching and temporary toxicity, highlighting the need for further optimization [73].

### 6.8. Bioaugmentation of Activated Sludge Systems

Previous studies have emphasized the potential of bioaugmentation with specialized microorganisms to enhance the removal of active pharmaceutical ingredients (APIs), particularly for recalcitrant compounds. In pure culture experiments, cometabolic degradation of zopiclone using skim milk powder as an additional nutrient source demonstrated the highest efficiencies with several microbial strains. The yeast *Apiotrichum domesticum* achieved >99% removal, while the bacteria *Pseudomonas putida* and *Moraxella osloensis* reached 85% and 89% removal, respectively, when used as sole carbon sources. Furthermore, filamentous fungi *Fusarium solani* and *Fusarium udum* both achieved >90% removal in the tests. These results highlight the high potential of bioaugmentation in improving the degradation of persistent Z-drugs. In particular, the bioaugmentation of activated sludge systems with these specialized microorganisms offers a promising approach to enhance the overall efficiency of wastewater treatment for recalcitrant pharmaceuticals, including Z-drugs [74,75].

### 6.9. Advanced Oxidation Processes (AOPs)

It should be emphasized that Advanced Oxidation Processes (AOPs) are widely recognized as highly effective techniques for removing pharmaceuticals from water due to their ability to generate highly reactive radicals, particularly hydroxyl radicals (·OH). Several AOPs have been extensively investigated, including ozonation, Fenton and photo-Fenton processes, UV-based methods (direct UV photolysis, UV/H_2_O_2_, UV/peracetic acid, UV/chlorine, UV/sulfate radicals), sonolysis (ultrasound), and electrochemical oxidation. Overall, removal efficiencies vary significantly depending on the specific AOP, the type and concentration of the pharmaceutical, the water matrix, and operating conditions such as pH, contact time, and catalyst dosage. Many studies report high to complete removal rates, often exceeding 90% for targeted pharmaceuticals. However, mineralization (TOC removal) is frequently lower, indicating the formation of by-products, which requires further investigation regarding their toxicity and environmental fate. Recent research trends focus on hybrid AOPs and the use of advanced materials, particularly TiO_2_-based photocatalysts, as previously stated, to improve both efficiency and sustainability in pharmaceutical removal [76].

### 6.10. Ecological Approaches Such as Algal Ponds and Constructed Wetlands

Another promising direction involves ecological approaches such as algal ponds and constructed wetlands, which offer low-cost and green alternatives, particularly when integrated with other advanced or conventional treatment methods in hybrid systems. Future research should therefore focus on optimizing such hybrid configurations to overcome individual limitations and enhance the overall efficiency of pharmaceutical removal from real wastewater [77]. Despite these advances and the intensive search for effective pharmaceutical removal methods, data specifically addressing the removal of Z-drugs remain very limited.

### 6.11. Factors Influencing the Removal Efficiency of Z-Drugs in WWTPs

Comparing removal efficiencies of Z-drugs across different wastewater treatment plants remains challenging due to substantial variations in the applied technologies, configurations, operational parameters, and microbial communities [21,70,74,75,78]. The treatment process itself is one of the primary determinants of pharmaceutical removal rates [70]. Differences in biological and operational design—such as activated sludge systems versus membrane bioreactors—lead to varying contact conditions between contaminants and microorganisms. Operational parameters including hydraulic retention time (HRT), sludge age, biomass concentration, temperature, and pH have all been identified as key factors influencing removal efficiency [74,75,78]. A longer HRT, for instance, enhances the interaction between the target compound and microbial biomass, typically resulting in higher degradation rates of pharmaceuticals [25]. Conversely, high influent loads or insufficient contact time may reduce removal efficiencies, particularly in plants with limited treatment capacity [70]. Similarly, seasonal changes that affect parameters such as temperature and dilution can reduce removal efficiency by limiting microbial activity and slowing biological processes, most notably in spring [61].

The composition and metabolic capabilities of microbial communities also play a decisive role. The removal rates of zopiclone and other Z-drugs differ significantly among microbial groups, suggesting that specific enzymatic pathways are crucial for their biotransformation. Many microorganisms lack the enzymes required to effectively degrade complex or stable pharmaceutical structures [74,75]. However, the presence of easily degradable co-substrates can enhance biodegradation through cometabolism. Studies have shown that when an additional nutrient source is available, cometabolic degradation markedly increases the removal efficiency of zopiclone compared with conditions where it serves as the sole carbon source. This indicates that the metabolic environment within a WWTP can strongly influence the overall fate of Z-drugs [74,75].

In addition to biological and operational factors, the physicochemical properties of the compounds themselves critically determine their removal behavior. Parameters such as molecular weight, solubility, hydrophobicity (log P), acid dissociation constant (pKa), and polarity (Table 1) directly affect processes such as sorption, volatilization, and biodegradation [21,24,26]. Highly soluble and polar compounds tend to remain dissolved in the aqueous phase and are therefore less susceptible to adsorption, whereas hydrophobic compounds with higher log P values are more readily adsorbed onto sludge particles, enhancing their apparent removal efficiency. Similarly, compounds with higher molecular weights often exhibit improved sorption and removal compared to lighter or more stable molecules [24,26].

Moreover, in WWTPs, pharmaceuticals may enter the treatment systems as parent compounds together with conjugated, oxidized, or otherwise transformed metabolites formed during human metabolism [79]. Deconjugation of these metabolites into their parent compounds through enzymatic activity and abiotic processes has been widely reported in sewage and WWTP effluents and is considered a possible explanation for observed negative removal rates [80]. For example, research has shown that conjugates of other organic compounds, such as steroid hormones, are readily deconjugated in domestic wastewater and in sewage treatment plants. This occurs due to the high levels of the enzyme β-glucuronidase, which is produced by the fecal bacterium *Escherichia coli*. It is therefore likely that glucuronide and sulfate conjugates of pharmaceutical compounds undergo degradation through the same mechanism [81].

Reported removal efficiencies of psychiatric pharmaceuticals varied widely, from negative values to nearly 100%, even for the same treatment process [70,81,82,83]. Removal was largely attributed to secondary treatment processes involving adsorption onto settled sludge and potential biodegradation. Facilities employing only conventional activated sludge processes were reported to exhibit lower removal efficiencies compared to systems including additional biological treatment units such as rotating biological contactors. High input loads, limited treatment capacity, and short contact times were also indicated as factors potentially reducing removal efficiency [70,84].

Pharmaceuticals with complex aromatic structures or high chemical stability typically show limited biodegradability, reflecting their design to resist biological transformation [25,26]. This resistance not only lowers biological removal efficiency but may also lead to the retransformation of metabolites back into parent compounds through deconjugation processes observed during treatment [70]. Furthermore, the potential of certain compounds to accumulate in sediments can contribute to partial removal via sedimentation mechanisms [26].

Overall, the efficiency of Z-drug removal in WWTPs results from a complex interplay of operational conditions, microbial activity, and compound-specific properties. Understanding these interdependencies is essential for optimizing treatment strategies and improving the removal of pharmaceutically active compounds from wastewater.

Incomplete removal of Z-drugs during wastewater treatment leads to their discharge as parent compounds, metabolites, and transformation products into surface and groundwaters. Apparent high removal efficiencies do not necessarily indicate complete degradation, as transformation products can depress measured concentrations, creating a false impression of effective removal. Although households, hospitals and the pharmaceutical industry are principal sources of these contaminants, wastewater treatment plants serve as a critical point for their release into the environment [61].

## 7. Fate of Z-Drugs in the Environment (Biodegradation, Sorption, Hydrolysis, Photolysis Data)

As mentioned above, Z-drugs are often incompletely removed by conventional wastewater treatment plants, and in some cases, concentrations may even increase after treatment due to negative removal. These compounds persist in the environment due to their resistance to standard treatment processes and can undergo various environmental transformations such as biodegradation, sorption to sediments, hydrolysis, and photolysis, which determine their stability and fate. However, literature data on this topic are very limited.

The environmental behavior of Z-drugs in natural waters and wastewaters might be influenced by their physicochemical properties, as summarized in Table 1. Although experimental data on Z-drugs are scarce, compounds with relatively low log P and high water solubility may tend to remain predominantly in the aqueous phase, which could potentially limit sorption onto sediments and suspended solids and facilitate transport in surface and groundwater [64]. This behavior could lead to wider spatial distribution in aquatic environments and might contribute to exposure of organisms across connected ecosystems [85]. Environmental fate studies suggest that pharmaceuticals undergo a range of biotic and abiotic transformation processes, including photolysis, microbial metabolism, and hydrolysis; however, the rates and dominant pathways appear to depend, often inferred from structurally similar compounds, on molecular structure and intrinsic stability [86]. Z-drugs, designed to be chemically stable, might degrade slowly under environmental conditions, and when combined with continuous input from wastewater effluents, this may result in pseudo-persistence, with compounds potentially remaining present despite partial degradation. Their water solubility and low sorption might promote continuous exposure of aquatic organisms, which could lead to chronic effects rather than classical bioaccumulation, depending on species-specific metabolic capacities. Overall, the combination of these properties—water solubility, moderate to low lipophilicity, and slow biodegradation—is likely to favor mobility and widespread occurrence of Z-drugs in aquatic environments, although direct experimental evidence for these patterns is currently limited.

The pharmacokinetic profiles summarized in Table 2 provide essential context for understanding the environmental fate of Z-drugs. Extensive human metabolism, high oral bioavailability, and predominantly urinary excretion of metabolites indicate that wastewater and surface waters are exposed largely to transformation products rather than parent compounds. Moreover, the metabolic pathways of Z-drugs differ among compounds; for instance, zolpidem is primarily metabolized by CYP3A4, whereas zaleplon is largely processed by aldehyde oxidase (Table 2). These differences result in distinct profiles of metabolites and transformation products, which may be more polar, less prone to sorption, and more resistant to biological removal. Consequently, observed environmental concentrations and persistence cannot be inferred solely from the physicochemical properties listed in Table 1, since the chemical form, mobility, and bioavailability of metabolites and degradation products may differ significantly from those of the parent drug. Likewise, the dose range and oral bioavailability (Table 2) directly influence the quantities of parent compounds and metabolites entering wastewater. As less than a few percent of the administered dose is excreted unchanged for most Z-drugs, influent concentrations of parent compounds may underestimate the actual pharmaceutical load entering WWTPs. Additionally, the presence of conjugated or labile metabolites may contribute to apparent variability in reported environmental fate, as these compounds can undergo deconjugation or transformation back to parent forms. However, experimental data on the environmental behavior, persistence, and ecotoxicity of these metabolites and transformation products remain scarce. This highlights the need to consider metabolites in environmental monitoring and risk assessment, even if their specific properties and transformation pathways are not fully characterized.

### 7.1. Zolpidem

#### 7.1.1. Biodegradation

Zolpidem is almost completely metabolized in the human body into inactive metabolites, with only trace amounts of the parent compound excreted in urine or feces [87]. Several transformation products of zolpidem have been identified, including zolpacid, oxozolpidem, zolpyridine, and zolpaldehyde [72,88]. In a mesocosm study investigating the degradation of 120 pharmaceuticals and their metabolites in wastewater over a 31-day period, zolpidem was classified as a Type-D compound—a recalcitrant drug metabolite—indicating that both the parent compound and its metabolites exhibited limited degradation under the tested conditions [89]. These findings suggest that zolpidem may persist in aquatic environments, which is crucial for understanding its environmental fate and potential ecological implications [74,90]. Consistent with this, zolpidem shows low biodegradability under standard test conditions, implying potential persistence in waters impacted by wastewater treatment plant effluents [74,90]. According to EPI Suite biodegradation models, zolpidem tartrate has a BIOWIN1 (linear probability model predicting rapid aerobic biodegradation) value of 0.97 and a BIOWIN5 (MITI linear model based on Japanese test data) value of 0.0098, suggesting also limited biodegradability under environmental conditions. The compound is classified as ‘potentially persistent’ (*p*), reflecting its tendency to resist microbial degradation [74]. Nevertheless, other studies have demonstrated that biological transformation, particularly under oxic conditions, represents a key mechanism of zolpidem removal in WWTPs, emphasizing the significant role of microbial activity in mitigating its environmental occurrence [67].

#### 7.1.2. Sorption

Sorption studies indicate that zolpidem exhibits a low affinity for sediments and organic matter, which increases its tendency to remain in the aqueous phase [90]. Consequently, only limited removal by sorption processes—typically not exceeding 25%—can be expected for this compound [67].

#### 7.1.3. Hydrolysis

Zolpidem exhibits considerable stability toward hydrolysis under typical environmental conditions. Hydrolysis under environmentally relevant pH (5–9) and temperature conditions appears negligible and does not significantly contribute to its removal from aquatic systems [88,90]. The compound is stable against neutral hydrolysis and thermal degradation, as no significant degradation of the active pharmaceutical ingredient (API) or drug product was observed in neutral solutions or in the solid state at 70 °C after 8 and 21 days [72]. It was found to remain stable for up to 72 h at room temperature in wastewater with a natural pH of 7.4. After 7 days, zolpidem degradation reached approximately 50% [55]. However, zolpidem tartrate shows instability toward hydrolysis under acidic and alkaline conditions, with degradation rates increasing at elevated temperatures [72]. Under both acidic and basic conditions, the same major hydrolytic degradation product, zolpacid, is formed via acid- or base-catalyzed cleavage of the amide moiety located on the lateral chain of the zolpidem structure [72]. Overall, these findings indicate that zolpidem remains relatively stable under typical environmental conditions but may undergo hydrolytic transformation under more extreme pH or temperature regimes.

#### 7.1.4. Photolysis

Photodegradation studies indicate that zolpidem is relatively resistant to direct photolysis under natural sunlight, suggesting that environmental photolysis is not a major degradation pathway for this compound [90]. The UV absorption maximum of zolpidem occurs at approximately 238 nm, which lies within the UV-B range of solar radiation [88], indicating potential, though limited, sensitivity to sunlight. Experimental data show that zolpidem tartrate in aqueous solution is susceptible to photolytic degradation, with approximately 10% of the active pharmaceutical ingredient (API) decomposing upon light exposure and forming several degradation products. In contrast, the API in solid form and in film-coated tablets exhibited negligible photolytic degradation, implying that the protective film layer effectively shields the drug from light-induced reactions. Photolysis initiates and accelerates the oxidative breakdown of zolpidem, producing higher quantities of degradation products than oxidation alone. The enamine moiety within the imidazopyridine ring is particularly photoreactive toward singlet oxygen, which may account for the formation of zolpyridine upon irradiation. Identified photodegradation products include oxozolpidem, zolpaldehyde, and zolpyridine [72]. Overall, these findings demonstrate that while zolpidem may undergo limited photodegradation in aqueous environments, its solid-state stability and relatively low susceptibility to sunlight indicate that photolysis is unlikely to represent a dominant environmental transformation process.

**Figure 6:**
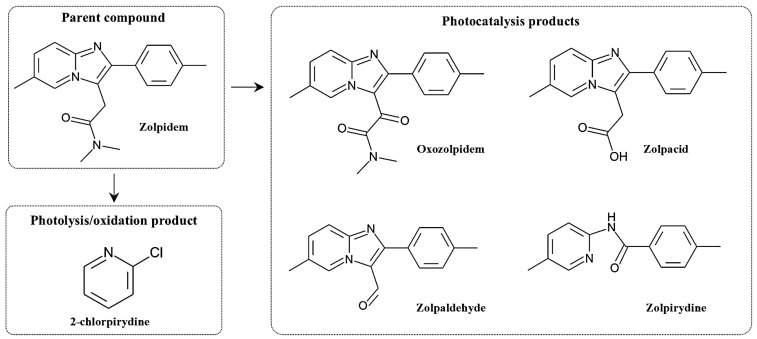
Proposed transformation products of zolpidem identified under tested degradation conditions [71,72,88].

Figure 6 summarizes the reported transformation products of zolpidem identified under tested degradation conditions [71,72,88], which represent the closest available approximation to environmentally relevant pathways. However, comprehensive data on transformation products of Z-drugs under true environmental conditions remain scarce, and most published studies rely on extreme or laboratory-specific stress conditions that are not directly representative of natural aquatic systems.

### 7.2. Zaleplon

Zaleplon exhibits very low solubility in water (Table 1), indicating limited mobility in aquatic environments and a potentially greater tendency to sorb onto solid particles such as sediments or sewage sludge. Its moderate partition coefficient (log P = 1.23) suggests only moderate hydrophobicity, implying a limited potential for bioaccumulation but a sufficient affinity for partial retention in the solid phase. Consequently, zaleplon may be distributed between the aqueous and sediment phases, with sorption likely reducing its bioavailability to microorganisms. The compound is also sensitive to light, which indicates the potential for photolytic degradation in surface waters [40]. However, hydrolysis appears to be the dominant degradation pathway, particularly under acidic conditions, where complete breakdown of the molecule has been observed [91]. Zaleplon undergoes extensive hepatic metabolism, with less than 1% of the parent compound excreted unchanged in urine (Table 2) [28]. The main metabolites, 5-oxo-zaleplon and desethylzaleplon, may also represent relevant transformation products in environmental matrices [40].

### 7.3. Zopiclone

#### 7.3.1. Hydrolysis

The environmental fate of zopiclone is governed primarily by chemical hydrolysis, which constitutes the dominant degradation pathway in aqueous media [92]. This reaction involves cleavage of the pyrrolidone ring, leading to the formation of 2-amino-5-chloropyridine (ACP) as the principal transformation product [92,93]. The reaction proceeds in a 1:1 stoichiometric ratio, indicating that ACP is formed exclusively from zopiclone, with no other major degradation pathways contributing significantly. The rate of hydrolysis is strongly influenced by environmental conditions, increasing with higher pH and temperature [92]. In aqueous solutions and nucleophilic solvents such as ethanol and methanol, zopiclone exhibits pronounced instability, which further confirms the central role of hydrolysis in its transformation [93]. The resulting ACP is pharmacologically inactive [94].

#### 7.3.2. Photolysis

Data on the photodegradation of zopiclone in natural environmental conditions are currently limited. However, laboratory studies indicate that zopiclone undergoes photodegradation when exposed to UVA irradiation. This process is influenced by the form of the drug and the presence of co-existing excipients. Zopiclone is susceptible to photodegradation, with decomposition rates varying between bulk substance and pharmaceutical preparations, depending on excipients such as titanium dioxide and talc. After 114 days of UVA exposure, zopiclone in its bulk form decomposed by 18.62%, while in pharmaceutical formulations, degradation reached 31.42%. This indicates that zopiclone generally decomposes faster in powdered tablets than in its pure bulk form. The proposed degradation pathways involve the cleavage of the pyrrolone ring, hydroxylation of the piperidine moiety, and the loss of the (4-methylpiperazinyl)carboxyl or chloropyridinyl fragments [95].

### 7.4. Eszopiclone

Data on the environmental fate of eszopiclone is limited, with no detailed studies on its biodegradation, sorption, hydrolysis, or photolysis. However, its extensive metabolism in humans—mainly through oxidation and demethylation, with less than 10% of the dose excreted unchanged (Table 2)—suggests that only small amounts of the parent compound reach the environment [96]. The reported instability of eszopiclone in biological matrices and aqueous media indicates potential for degradation under environmental conditions. Its degradation follows pseudo-first-order kinetics, is temperature-dependent, and shows a half-life of 231 h at 25 °C [97], implying moderate persistence and the need for further environmental studies.

## 8. Presence of Z-Drugs in Environmental Matrices (Monitoring Data)

Z-drugs are increasingly being detected in aquatic environments as residues resulting from therapeutic use. Compounds from this group and their metabolites have been detected in various environmental matrices worldwide. Literature data indicate their presence primarily in influent and effluent wastewater, but also in surface water, sediments, and coastal waters, although the concentrations measured are usually low, in the ng L^−1^ level (Table 5). Their presence, especially in wastewater and surface waters, has attracted growing attention due to potential environmental risks. Additionally, the analysis of these compounds in wastewater is applied in wastewater-based epidemiology (WBE) [21,27,33,56], a tool that enables the estimation of psychoactive substance consumption within a given population. This chapter provides an overview of literature data on the occurrence of Z-drugs and their metabolites in various environmental matrices. To date, no published data are available regarding the occurrence of non-metabolic transformation products in environmental matrices.

### 8.1. Wastewater

Z-drugs are frequently detected in wastewater, reflecting their widespread use and incomplete removal during conventional treatment. Concentrations vary significantly across regions, highlighting differences in consumption patterns and treatment efficiency.

#### 8.1.1. Occurrence of Z-Drugs in Wastewater

Among the parent compounds, zolpidem is the most commonly reported. In North America, concentrations reached up to 67 ng L^−1^ in Arizona (USA), representing the highest value among American studies, while data from Mexico indicate non-detectable levels. Zaleplon showed average concentrations of 6 ng L^−1^ in the USA and 8 ng L^−1^ in Mexico [27]. In the USA, zopiclone was not detected in either influent or effluent samples [32].

In Asia, the highest concentrations of zolpidem were reported in Taiwan, ranging from 2–3388 ng L^−1^ with a detection frequency (DF) ranging from 56% to 100% [52], representing the maximum value among all reviewed studies. In Beijing, influent levels reached up to 23 ng L^−1^, while effluent concentrations were slightly higher, up to 33 ng L^−1^ [25]. In Busan (South Korea), influent concentrations ranged from 2.6–4.9 ng L^−1^ (DF = 100%), and effluent levels were generally lower, between 0.9 and 2.0 ng L^−1^ [24].

In Europe, concentrations of zolpidem were generally lower than in Asia, with the highest level reported in the Czech Republic, ranging from not detected to 52 ng L^−1^ [26]. Two studies from Spain indicated lower concentrations: one reported influent levels up to 7 ng L^−1^ (DF = 71) [57], while another found influent concentrations ranging from not detected to 3.9 ng L^−1^ and effluent concentrations from not detected to 7.1 ng L^−1^ [55]. In Portugal, zolpidem was not detected [61]. In the UK, influent samples showed zolpidem concentrations ranging from not detected to 1.1 ng L^−1^, with no effluent data available, while enantiomer-specific analysis for zaleplon in the same study revealed that none of the target analytes were detected [53].

#### 8.1.2. Occurrence of Metabolites of Z-Drugs in Wastewater

Metabolites of Z-drugs have been analyzed less frequently, but when included, they can occur at relatively high concentrations. In Asia, zolpidem 4-phenylcarboxylic acid reached relatively high concentrations in Busan (South Korea), with influent levels of 65.5–237.1 ng L^−1^ and effluent levels of 62.4–116.8 ng L^−1^ (DF = 100) [24]. Complementary data from another Korean study, based on non-target screening, indicated semi-quantitative values ranging from 6.75 to 39.3 ng L^−1^ [59]. These findings highlight the persistence of transformation products even when parent compounds are partially removed, emphasizing the need for comprehensive monitoring strategies.

### 8.2. Surface Water

Z-drugs have been detected in surface waters, including rivers, lakes, and coastal marine environments. In freshwater systems, most studies focused on rivers. In the Seine River (France), zolpidem concentrations ranged from LOQ to 0.28 ng L^−1^ (DF = 100), while zopiclone reached up to 3.51 ng L^−1^ (DF = 89) [23]. Among metabolites, zolpidem 4-phenylcarboxylic acid was found at levels up to 8.5 ng L^−1^, and zolpidem 6-carboxylic acid up to 1.01 ng L^−1^, whereas zopiclone N-oxide was not detected [23]. In the Danube River (Hungary), zolpidem concentrations ranged from 0.02 to 0.62 ng L^−1^ (DF = 18.7) [54]. In Spain, zolpidem was not detected in a river located 5 km downstream from a WWTP [55]. One study investigated a lake in New York State (USA), where zopiclone was not detected [32].

In marine environments, Z-drugs have also been detected, although concentrations remain in the low ng L^−1^ range. Along the North Atlantic Portuguese coast, zolpidem was reported at levels between 0.3 and 4.2 ng L^−1^ (DF = 100) [62], representing the highest concentrations observed in coastal waters (Table 4). In the southern coast of Viti Levu (Fiji, South Pacific), concentrations ranged from 0.78 to 2.6 ng L^−1^ (DF = 1) [99], while in northwestern Spain, values varied from non-detectable to 1.46 ng L^−1^ [98]. These findings indicate that zolpidem persists even in marine environments, with concentrations influenced by proximity to wastewater discharge points and regional hydrodynamics.

### 8.3. Drinking Water

Two studies investigated Z-drugs in drinking water (likely groundwater sources). In one study, zopiclone was not detected [32], while in the other, zolpidem was found at 0.04 ng L^−1^ (DF = 1.1) [54]. Overall, concentrations in drinking water were considerably lower than in wastewater or surface waters.

### 8.4. Sediments

Only one study reported the presence of zolpidem in sediments, without quantitative data [58]. This indicates that sediments are poorly investigated for Z-drugs.

### 8.5. Biota

Environmental monitoring of biota is extremely limited. Only one study analyzed fish tissues, reporting zolpidem levels below the limit of quantification (<LOQ) [62], suggesting low bioaccumulation potential and very limited data availability.

### 8.6. Concluding Remarks

Overall, based on available data, the concentrations of Z-drugs and their metabolites reported in environmental matrices are generally low and do not exceed predicted no-effect concentrations (PNEC) for acute toxicity. For example, PNEC values for zolpidem 4-phenylcarboxylic acid range from 4690 ng L^−1^ for algae to 42,800 ng L^−1^ for *Daphnia* [21], suggesting a low ecotoxicological risk for individual compounds. Furthermore, the available data remain scarce and inconsistent, with significant gaps in monitoring, especially for sediments, biota, and certain compounds such as eszopiclone. Most existing monitoring studies quantify Z-drugs as racemates, only one study has attempted enantioselective analysis in wastewater, and no enantiomers were detected [53]. Available pharmacokinetic data show that zopiclone displays stereoselective behavior in vivo, with higher levels of the S-enantiomer (eszopiclone) [100]. This suggests that potential differences between enantiomers cannot be excluded also in environmental samples, but current monitoring approaches are not able to capture them. Therefore, future studies should consider enantioselective analytical methods to better assess the occurrence and fate of individual enantiomers. The lack of standardized analytical procedures further complicates comparisons between studies. Continued research is therefore essential to improve occurrence data and develop effective strategies for environmental risk management.

## 9. Ecotoxicological Effects

The incomplete elimination of Z-drugs in WWTPs contributes to their occurrence in surface waters, where they may accumulate and potentially exert ecotoxicological effects [21,73]. Although many pharmaceuticals do not exhibit acute toxicity at environmentally relevant concentrations, their capacity for bioaccumulation and the consequences of chronic, low-dose exposure on non-target organisms remain poorly understood [58]. It should be noted that pharmaceutical pollutants can significantly affect aquatic organisms by altering their behavior, physiology, and reproduction.

Z-drugs, in turn, classified as sedative–hypnotic pharmaceuticals, directly affect the central nervous system by modulating GABA___A receptors to induce sleep and relaxation (Table 2) [25,55,101]. Consequently, their presence in aquatic environments may pose risks to non-target species, with prolonged exposure potentially resulting in chronic ecological and evolutionary effects. Although Laimou-Geraniou et al. reported that environmental assessments did not predict acute effects on aquatic organisms for the measured concentrations of several antipsychotic and benzodiazepine residues, the authors also highlighted reports of high environmental risk (RQ > 1) for the antipsychotics clozapine and risperidone, indicating their persistence, bioaccumulation potential, and toxicity [21]. In this context, Z-drugs, which share similar neuroactive properties with benzodiazepines, also warrant careful environmental risk assessment.

Neuroactive substances such as Z-drugs are of particular concern, as they can bioaccumulate in fish tissues and interfere with neurological, reproductive, and behavioral processes. These sublethal effects may lead to population-level disturbances and long-term alterations in aquatic ecosystem dynamics [62]. Ecotoxicological predictions, such as those generated by ECOSAR models, indicate that the levels of Z-drugs detected in surface waters may pose a potential threat to aquatic organisms and can reach concentrations that are toxic to fish and invertebrates. It is worth noting that some of these compounds exhibit high distribution factors (Df > 80%), suggesting their considerable potential for environmental dispersion and persistence [21]. Preliminary environmental risk assessment models indicate the need to account for behavioral changes, bioaccumulation potential, and synergistic interactions with other co-occurring pharmaceuticals in water [51]. Furthermore, transformation products formed during treatment processes or in natural waters may possess ecotoxicological properties that are not yet fully characterized, emphasizing the necessity for further investigation [23].

Importantly, the pharmacokinetic profiles summarized in Table 2 indicate that Z-drugs are extensively metabolized in humans, with only a minor fraction excreted unchanged, while the majority is released as metabolites. Consequently, aquatic environments receiving WWTP effluents are likely exposed not only to parent Z-drugs but also to a complex mixture of human metabolites and transformation products. Although these compounds may differ substantially in polarity, persistence, and bioavailability compared to the parent substances, experimental data on their ecotoxicological effects are largely lacking. This gap limits current risk assessments, as the contribution of metabolites and environmental transformation products to observed sublethal or chronic effects in aquatic organisms remains uncertain. Moreover, the co-occurrence of parent Z-drugs, their metabolites, and transformation products in aquatic environments raises concern that combined exposure may result in additive or potentially synergistic ecotoxicological effects, even when individual compounds occur at low concentrations. Overall, the limited ecotoxicological information available for Z-drugs (Table 6) emphasizes the necessity of further research into their potential long-term risks to aquatic organisms.

Based on the physicochemical properties summarized in Table 1, including chemical stability, low sorption potential, and moderate to high water solubility, Z-drugs may persist in aquatic environments for extended periods due to their continuous release from wastewater treatment plants. Even when partial biodegradation occurs, it may lead to the formation of more mobile transformation products rather than complete mineralization. This sustained presence raises concerns regarding long-term exposure of aquatic organisms; however, experimental and literature data on the ecotoxicological effects of specific environmental transformation products and metabolites of Z-drugs remain largely unavailable. To date, ecotoxicity information is reported almost exclusively for the parent compounds, with zolpidem 4-phenylcarboxylic acid, referenced in Table 6, being one of the few metabolites for which environmental relevance has been discussed. The absence of systematic ecotoxicological data for Z-drug transformation products represents a significant knowledge gap and currently limits comprehensive environmental risk assessment.

Importantly, environmentally relevant exposure scenarios for Z-drugs are characterized not by short-term peak concentrations but by continuous, low-level exposure resulting from their incomplete removal in WWTPs and sustained release into receiving waters. Under such conditions, chronic and sublethal effects [102], including neurobehavioral alterations, impaired reproduction, and changes in predator–prey interactions, may be ecologically more relevant than acute toxicity endpoints [103,104]. Moreover, Z-drugs co-occur in aquatic environments with other neuroactive pharmaceuticals, raising concerns regarding additive or synergistic effects that are not captured by single-compound toxicity assessments. The potential contribution of metabolites and transformation products to mixture toxicity further complicates the evaluation of long-term ecological risks.

It should be emphasized that the complexity of real environmental exposure scenarios, involving chronic low-dose exposure, mixture effects, and variable environmental conditions, makes ecological risk assessment highly uncertain, and the currently available experimental data for Z-drugs are insufficient to draw robust and unambiguous conclusions regarding their long-term ecotoxicological impacts.

### 9.1. Zolpidem

Zolpidem undergoes extensive metabolic transformation in humans, resulting in limited excretion of the parent compound into the environment. The ecotoxicity data presented in Table 6 indicate that zolpidem exhibits acute toxicity toward aquatic organisms, with algae showing the highest sensitivity (LC50 = 2.2 mg L^−1^) compared to *Daphnia* and fish [87]. Its main metabolite, zolpidem 4-phenylcarboxylic acid (ZPCA) (Figure 3), generally displays higher LC50 and NOEC values, suggesting lower toxic potential. Although zolpidem shows measurable toxicity, particularly toward freshwater algae, the relatively low levels released and limited environmental persistence suggest that the overall ecological risk is likely low. Nevertheless, species-specific sensitivities and potential chronic effects should be considered in future environmental risk assessments.

The phytotoxicity and biostimulant effects of zolpidem were evaluated using plant biotests with agriculturally relevant species (*Raphanus sativus*, *Lactuca sativa*, and *Triticum aestivum*). The results are summarized in Table 7 [105].

*Raphanus sativus* (radish) was used in a preliminary phytotoxicity assay to evaluate the acute toxicity and phytotoxic effects of zolpidem during germination. Chosen for its rapid growth and sensitivity to pollutants, this species serves as an effective bioindicator for pharmaceutical contaminants. The study revealed that the phytotoxic effects of zolpidem are likely linked to its accumulation, or that of its metabolites, in root tissues, impairing germination and root development. The responses observed follow a hormetic pattern, where low concentrations exert neutral or mildly stimulatory effects, while higher concentrations induce adverse outcomes beyond a specific threshold. Moderate doses (e.g., 10 mg L^−1^) enhanced plant performance, promoting leaf greenness, root elongation, and fresh biomass, whereas higher concentrations (15–20 mg L^−1^) caused phytotoxicity, particularly in lettuce, highlighting a clear dose-dependent effect. Species-specific differences were noted, with dicotyledonous lettuce showing a stronger stimulation of shoot development [105]. These results emphasize the need for monitoring zolpidem in agricultural soils and wastewater and for establishing safe environmental concentration guidelines.

### 9.2. Zaleplon

Although no direct aquatic toxicity studies have been conducted for zaleplon (fish, algae, or *Daphnia*, Table 6), its mechanism of action on GABA_A receptors raises potential concerns. Similar receptors are present in aquatic organisms, suggesting that zaleplon could exert neuroactive effects in fish and invertebrates [40].

### 9.3. Zopiclone

Zopiclone undergoes hydrolysis under both acidic and alkaline conditions, producing distinct degradation products. Acidic hydrolysis yields 6-(5-chloro-2-pyridyl)-7-hydroxy-6,7-dihydro-5H-pyrrolo [3,4-b]pyrazine-5-one, while alkaline hydrolysis produces 2-amino-5-chloropyridine. Chloropyridine derivatives are relatively stable under environmental conditions and may be toxic to aquatic organisms, reflecting the persistence and bioactivity commonly associated with chlorinated pyridines. Hydroxylated pyrazinone derivatives are more polar, potentially less bioaccumulative, and more water-soluble; however, they may still interact with biological systems. Long-term exposure or release into basic waters favors conversion to chloropyridine, which resists further degradation, whereas acidic or neutral hydrolysis products degrade more slowly, highlighting potential environmental persistence [94].

Hydrolysis of zopiclone analogues results in products that are pharmacologically inactive, as evidenced by in vitro binding assays to the benzodiazepine receptor (IC_50_ > 1000 nM). Mechanistically, hydrolysis cleaves the piperazinyl carbamate moiety, which is essential for receptor binding and pharmacological activity. Consequently, these hydrolysis products are less likely to exert receptor-mediated effects in aquatic organisms if released into the environment. Nevertheless, the chemical stability of these compounds in water or soil may still pose environmental concerns, particularly for analogues that resist hydrolysis under acidic conditions [94].

Zopiclone is classified as potentially persistent and can bioaccumulate, although specific ecotoxicological effects are not well-documented. Its limited removal in WWTPs contributes to environmental persistence. The widespread presence of human and veterinary active pharmaceutical ingredients (APIs) in aquatic environments is a growing concern, as these substances may lead to unpredictable ecological impacts, including adverse effects in aquatic vertebrates and invertebrates. Potential outcomes include behavioral and reproductive disturbances, as well as feminization in fish, reflecting broader concerns associated with persistent neuroactive pharmaceuticals in surface waters [94].

### 9.4. Eszopiclone

Chiral pharmaceuticals, including both racemic mixtures and enantiomerically pure forms, along with their metabolites, can enter aquatic environments through wastewater effluents from inefficient treatment processes or via direct human disposal. Even at low concentrations, these compounds may adversely affect nontarget organisms, underscoring the importance of assessing the ecotoxicity of both enantiomers for accurate environmental risk evaluation [22]. Eszopiclone, a psychoactive drug, requires precise quantification in biological samples; however, it is highly prone to rapid degradation during specimen handling. Notably, eszopiclone can convert into 2-amino-5-chloropyridine (ACP), a hydrolysis product, which may lead to underestimation of its actual concentration and inaccuracies in pharmacokinetic (PK) parameters. Both eszopiclone and its metabolites are unstable when exposed to room temperature or alkaline conditions, facilitating ACP formation [106]. Although direct ecotoxicological data for ACP are scarce, chlorinated pyridine derivatives are generally persistent and potentially bioactive [107], highlighting the need for careful monitoring of eszopiclone and its transformation products in aquatic systems.

In conclusion, Z-drugs, their metabolites and transformation products are recognized as persistent emerging contaminants in aquatic environments, posing potential ecotoxicological risks through bioaccumulation, interference with biological systems, and neuroactive effects on non-target organisms. While some, like zolpidem, may have a low overall ecological risk due to limited environmental presence, the general lack of comprehensive ecotoxicological data, particularly for chronic and sublethal effects, highlights the critical need for further research and robust environmental risk assessments for these compounds and their transformation products.

## 10. Summary

**Figure 7:**
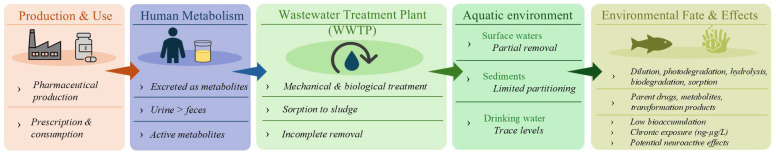
Schematic representation of the complete environmental life cycle of the studied pollutants, from production and human metabolism to wastewater pathways, treatment, and environmental fate.

Figure 7 provides a schematic overview of the complete environmental life cycle of the studied pollutants, from their production and human metabolism to wastewater transport, treatment, and eventual fate in aquatic ecosystems. Z-drugs are important therapeutic tools in insomnia management; however, evolving evidence challenges their initially presumed safety advantages over benzodiazepines [108,109]. Current regulatory frameworks increasingly integrate pharmacovigilance, abuse potential, sex-specific dosing considerations, and environmental risk assessment. Technological optimization strategies—ranging from precision dosing and innovative formulations to green pharmaceutical design—offer pathways to enhance safety, reduce misuse, and mitigate environmental impact [110,111].

This review paper presents the current state of knowledge on the occurrence of Z-drugs, their metabolites and transformation products in the environment. It addresses key topics including the physicochemical and pharmacokinetic properties of Z-drugs, their consumption levels, analytical methods used for determining Z-drugs and their major metabolites in environmental samples, the efficiency of their removal in wastewater treatment plants, and their environmental fate (biodegradation, sorption, hydrolysis, and photolysis). In addition, the occurrence of Z-drugs in various environmental matrices and their ecotoxicological effects are discussed. This paper summarizes and critically evaluates the available data on these topics.

## 11. Conclusions and Future Perspectives

Available data on analytical techniques for detecting Z-drugs and their major metabolites in environmental samples, their removal efficiency in wastewater treatment plants, their environmental fate, occurrence in various matrices, and associated ecotoxicological impacts remain scarce. Although households, hospitals and the pharmaceutical industry are principal sources of these contaminants, wastewater treatment plants serve as a critical point for their release into the environment. Incomplete removal of Z-drugs during wastewater treatment leads to their discharge as parent compounds, metabolites, and non-metabolic transformation products to water. Transformation during treatment or in natural waters can also produce intermediate products, whose formation and behavior require further investigation. Significant research gaps still exist regarding the analytical procedures, WWTP systems, monitoring data, environmental fate, transformation pathways, and long-term impacts of Z-drugs in aquatic systems. In particular:

(1) No single analytical method is universally optimal, underscoring the need for continued methodological refinement, the routine use of isotopically labelled internal standards, and greater harmonization of analytical protocols. Furthermore, significant challenges persist in metabolite (lower recoveries and reduced ionization efficiency) and non-metabolic transformation products detection. There is also a clear need to implement enantioselective analytical techniques to accurately characterize the ecological exposure and behavior of individual isomers.
(2) The removal efficiencies of Z-drugs and their metabolites in wastewater treatment plants remain inadequate and require improvement. This need is closely linked to the new Directive (EU) 2024/3019 of the European Parliament and of the Council on urban wastewater treatment, adopted on 27 November 2024 [112]. Consequently, greater emphasis should be placed on developing effective technologies for eliminating Z-drugs and their metabolites from surface waters and wastewater.
(3) Most monitoring studies to date have quantified Z-drugs as racemic mixtures. Only one investigation has attempted enantioselective analysis in wastewater [53]. Pharmacokinetic evidence demonstrates that zopiclone exhibits stereoselective behavior in vivo, with higher concentrations of the S-enantiomer (eszopiclone) [100]. This finding suggests that enantiomer-specific differences may also occur in environmental matrices. Thus, future research should therefore integrate enantioselective analytical techniques to enable a more accurate assessment of the occurrence, distribution, and environmental fate of specific enantiomers.
(4) There is a lack of comprehensive data from standardized OECD tests (e.g., biodegradability, adsorption/desorption, hydrolysis, and photolysis assays), which are essential for assessing their environmental persistence. Consequently, knowledge regarding the non-metabolic transformation products, their environmental occurrence, and their ecotoxicity remains extremely limited. This lack of data highlights the need to consider Z-drugs as emerging contaminants and justifies further research on their occurrence, behavior, and ecological risks.
(5) The effects of Z-drugs on microbial ecosystems remain largely uncharted, representing a significant knowledge gap in environmental toxicology. The continuous release of these compounds and their metabolites into aquatic environments raises concerns about their potential ecotoxicological impacts and highlights the need for further research into their comprehensive environmental fate and removal strategies.
(6) Z-drugs occur in aquatic environments together with other neuroactive pharmaceuticals, raising concerns about potential additive or synergistic interactions that cannot be adequately captured by single-substance toxicity assessments. The presence of metabolites and transformation products—each of which may contribute to overall mixture toxicity—further complicates the evaluation of ecological risks and underscores the need for more comprehensive investigation.
(7) Future regulatory approaches should adopt a more holistic, data-driven, and sustainability-oriented model to balance therapeutic benefit with public health and environmental protection.

In summary, given the global occurrence of sleeping pill contamination, further comprehensive research is needed to elucidate the effects of Z-drugs on environmental (micro)organisms and to assess their broader ecological consequences.

## Figures and Tables

**Table 3:** Comparative table of analytical approaches for Z-drug analysis in environmental samples.

Analytes	Matrix	Analytical Technique	Mass Analyzer	Ionization	Column	Mobile Phase	Elution	Sample Pretreatment	Ref.
Zolpidem, Zopiclone (among 26 drugs)	Wastewater	LC-MS/MS	QqQ ^a^	ESI	Kinetex Biphenyl 2.6 µm, 100A (100 mm × 2.1 mm) ^b^	A: water + 0.1% HCOOH B: water/MeOH (1/9, *v*/*v*) + 0.1% HCOOH	Gradient	SPE	[33]
Zolpidem (among 114 organic micropollutants)	Surface water	LC-MS/MS	QTRAP	ESI	Kinetex F5 100A 2.6 µm (50 mm × 3.0 mm) ^b^	A: water + 0.1% HCOOH B: ACN	Gradient	SPE	[56]
Zolpidem (among 68 abused drugs)	Wastewater	LC-MS/MS	QqQ	ESI	Restek Ultra PFPP 5 µm (50 mm × 2.1 mm) ^c^	A: water + 0.1% HCOOH + 2% MeOH B: MeOH + 0.1% HCOOH	Gradient	DLLME	[52]
Zolpidem (nontarget analysis)	Freshwater sediments	UHPLC-MS/MS	QTOF	ESI	Acquity BEH C18 1.7 µm (50 mm × 2.1 mm) ^d^	A: water (for NI ^e^) (water + 0.1% HCOOH for PI ^f^) B: ACN (for NI) (ACN + 0.1% HCOOH for PI)	Gradient	Soxhlet extraction + fractionation on silica gel	[58]
Zolpidem Zolpidem 4-phenylcaboxylic acid (nontarget analysis)	Wastewater	UHPLC-MS/MS	Q-Orbitrap ^g^	HESI ^h^	Acquity BEH C18 1.7 µm (50 mm × 2.1 mm) ^d^	A: water + 0.1% HCOOH B: MeOH + 0.1% HCOOH	Gradient	SPE	[59]
Zolpidem (among 3 drugs)	Surface water and wastewater	Fluorescence Spectroscopy	N/A ^i^	N/A	N/A	N/A	N/A	Filtration and pH adjustment	[60]
Zolpidem E1-Zopiclone E2-Zopiclone (R/S-enantiomers) (analysis of enantiomers among 56 drugs)	Wastewater	LC-MS/MS	QqQ	ESI	CHIRALPAK CBH 5 µm (100 mm × 2.0 mm) ^j^	A: 1 mM CH_3_COONH_4_ (85%) B: MeOH (15%)	Isocratic	SPE	[53]
Zolpidem (among 25 other drugs and personal care products)	Wastewater	UHPLC-MS/MS	QqQ	ESI	Acquity BEH C18 1.7 µm (100 mm × 2.1 mm) ^d^	A: water + 0.01% HCOOH B: MeOH	Gradient	Centrifugation	[26]
Zolpidem, zolpidem 4-phenylcaboxylic acid, zolpidem 6-carboxylic acid, zopiclone, zopiclone-N-oxide (among 68 drugs)	Surface water	LC-MS/MS	QqQ	ESI	Acquity BEH C18 1.7 µm (100 mm × 2.1 mm) ^d^	A: water + 0.1% CH_3_COOH B: ACN + 0.1% CH_3_COOH	Gradient	SPE	[23]
Zolpidem (among 37 psychoactive substances)	Wastewater	LC-MS/MS	QTRAP	ESI	Purospher Star RP-18 5 µm (125 mm × 2.0 mm) ^k^	A: 5 mM HCOOH/HCOONH_4_ B: ACN	Gradient	On-line SPE	[57]
Zolpidem (among 52 pharmaceuticals)	Surface water	SFC-MS/MS	QqQ	Not specified	Acquity UPC2 BEH 1.7 µm (100 mm × 2.1 mm) ^d^	Not specified	Not specified	SPE	[54]
Zolpidem (among 33 neuroactive pharmaceuticals)	Surface water and fish tissues	LC-MS/MS	QqQ	HESI	Hypersil gold	Not specified	Not specified	Water: SPE Fish tissue: Extraction with ACN including tissue disruption with zirconium beads and centrifugation	[62]
Zolpidem (among 23 drugs)	Wastewater and surface water	LC-MS/MS	QqQ	ESI	Synergi Fusion RP 4 µm (100 mm × 2.0 mm) ^b^	A: water + 0.1% CH_3_OOH B: MeOH + 0.1% CH_3_OOH	Gradient	SPE	[55]
Zolpidem (among 11 drugs)	Wastewater	LC-MS/MS	Ion trap	ESI	Pursuit UPS C18 2.4 µm (50 mm × 2.1 mm) ^l^	A: 10 mM HCOOH (aq.) B: MeOH	Gradient	SPE	[61]
Zaleplon (among 22 psychiatric drugs)	Wastewater	LC-MS/MS	QqQ	ESI	X-bridge C18 3.5 µm (150 mm × 2.0 mm) ^d^	A: water + 0.2% HCOOH (pH 3.5) B: ACN/MeOH (50:50, *v*/*v*)	Gradient	SPE	[25]

^a^ Triple quadrupole; ^b^ Phenomenex, Torrance, CA, USA; ^c^ Restek, Bellefonte, PA, USA; ^d^ Waters, Milford, MA, USA; ^e^ Negative ionization; ^f^ Positive ionization; ^g^ Quadrupole-Executive Orbitrap; ^h^ Heated ESI; ^i^ not available; ^j^ Chiral Technologies, Illkirch, France; ^k^ Merck, Darmstadt, Germany; ^l^ Varian, Palo Alto, CA, USA.

**Table 4:** Comparison of wastewater treatment technologies investigated for the removal of Z-drugs and their metabolites based on reported literature data.

Treatment Approach (Section)	Target Z-Drug (s)	Reported Removal Efficiency	Dominant Mechanism(s) Reported	Transformation Products Reported	Study Scale/Conditions (as Stated)	Ref.
CAS + rotating biological contactor (Section 6.1)	Zolpidem	up to 100%	biodegradation/adsorption	not specified	full-scale WWTP	[70]
Enhanced CAS with additional processes (Section 6.2)	Zolpidem, zolpidem-4-phenylcarboxylic acid	56.4% (parent), 10.1% (metabolite)	adsorption + biological degradation	not specified	full-scale WWTP	[24]
AGS vs. CAS (Section 6.3)	Zolpidem	CAS > 50%, AGS > 75% residual	aerobic biodegradation	not specified	lab & full-scale	[67]
MBR/MBBR/AS (Section 6.4)	Zolpidem, zolpidem–phenylcarboxylic acid	11–81% (parent), –2–74% (metabolite)	biological treatment	not specified	full-scale WWTP	[21]
Photocatalysis (Sn–N–TiO_2_) (Section 6.5)	Zopiclone	up to 91%	photocatalytic degradation	2-chloropyridine	lab-scale	[71]
Light stress/extreme conditions (Section 6.6)	Zolpidem	significant only under extreme conditions	photolysis/oxidation	zolpacid, oxozolpidem, zolpyridine, zolpaldehyde	stress tests	[72]
Adsorption (MPB, BC, AC) (Section 6.7)	Zopiclone	6.7–96.7%	adsorption	not specified	batch & pilot-scale	[73]
Bioaugmentation (Section 6.8)	Zopiclone	>85–99%	microbial biodegradation	not specified	pure cultures	[74,75]
AOPs (Section 6.9)	Z-drugs (general)	often >90%	radical oxidation	not specified	lab-scale	[76]
Algal ponds/wetlands (Section 6.10)	Z-drugs (general)	not quantified	ecological uptake	not specified	conceptual/pilot	[77]

**Table 5:** Reported data of Z-drugs and their metabolites in environmental matrices.

Compound	Country/ Region	Sampling Season	Concentration Range (Mean) Water Samples [ng L^−1^]/Sediment/Fish Tissue [ng g^−1^]							Analytical Technique	Ref.
			Wastewater		Surface Water	Drinking Water	Coastal Water	Sediments	Fish Tissue		
			Influent	Effluent							
Zolpidem	South Korea (Busan)	April 2018	2.6–4.9 (3.6) DF ^a^ = 100	0.9–2.0 (1.4) DF = 100	-	-	-	-	-	SPE-LC-MS/MS	[24]
	Taiwan (Taipei)	September 2021–January 2024	2-3388 DF = 56–100	- ^b^	-	-	-	-	-	DLLME-LC-MS/MS	[52]
	Spain (Rias Baixas)	July 2015	-		-	-	n.d.–1.46	-	-	LC-MS/MS	[98]
	Fiji (Viti Levu)	Wet summer, dry winter	-		-	-	0.78–2.6 (1.7) DF = 1	-	-	SPE-LC-MS/MS	[99]
	USA (10 states)	July–October 2020	n.d. ^c^–67		-	-	-	-	-	SPE-LC-MS/MS	[27]
	Mexico (14 states)		n.d.								
	Hungary (Budapest)	Summer 2017, Spring 2018, Summer 2018, Autumn 2018/2019	-		0.02–0.62 (0.28) DF = 18.7	(0.04) DF = 1.1	-	-	-	SFC-MS/MS	[54]
	Portugal	Spring and summer 2013	n.d.		-	-	-	-	-	SPE-LC-MS/MS	[61]
	China (Beijing)	August–Sepember 2011	n.d.–23 ± 7	n.d.–33 ± 1	-	-	-	-	-	SPE-LC-MS/MS	[25]
	Spain (Barcelona)	March 2015	7.0 DF = 71		-	-	-	-	-	On-line-SPE- LC-MS/MS	[57]
	Croatia, Zagreb	February 2008	-		-	-	-	detected	-	LC-MS/MS (nontarget)	[58]
	UK	Not specified	n.d.–1 ± 1	-	-	-	-	-	-	SPE-LC-MS/MS	[53]
	France (Seine River)	Not specified	-		<LOQ ^d^–0.28 (0.18) DF = 100	-	-	-	-	SPE-LC-MS/MS	[23]
	Portugal (Duoro, Tejo, Sado, Mira rivers)	Summer 2019	-		-	-	0.3–4.2 DF = 100	-	<LOQ	LC-MS/MS	[62]
	Spain	Not specified	n.d.–3.9	n.d.–7.1	n.d.	-	-	-	-	SPE-LC-MS/MS	[55]
	Czech Republic	April–May 2020	n.d.–52.0		-	-	-	-	-	SPE-LC-MS/MS	[26]
Zaleplon	USA (10 states)	July–Oct.2020	6 ± 1		-	-	-	-	-	SPE-LC-MS/MS	[27]
	Mexico (14 states)		8 ± 1								
Zopiclone	USA (NY state)	Not specified	n.d.	n.d.	n.d.	n.d.	-	-	-	SPE-LC-MS/MS	[32]
	France (Seine River)	Not specified	-		<LOQ–3.51 (2.45) DF = 89	-	-	-	-	SPE-LC-MS/MS	[23]
E1-Zopiclone E2-Zopiclone	UK	Not specified	n.d.	-	-	-	-	-	-	SPE-LC-MS/MS	[53]
Zolpidem 4-phenylcarboxylic acid	South Korea (Busan)	April 2018	65.5–237.1 (105.7) DF = 100	62.4–116.8 (79.5) DF = 100	-	-	-	-	-	SPE-LC-MS/MS	[24]
	South Korea	May 2021	6.75–39.3 ^e^ (18.5) ^e^ DF = 100		-	-	-	-	-	SPE-LC-MS/MS (nontarget)	[59]
	France (Seine River)	Not specified	-		<LOQ–8.5 (3.75) DF = 100	-	-	-	-	SPE-LC-MS/MS	[23]
Zolpidem 6-caboxylic acid	France (Seine River)	Not specified	-		<LOQ–1.01 (0.63) DF = 53	-	-	-	-	SPE-LC-MS/MS	[23]
Zopiclone-N-oxide	France (Seine River)	Not specified	-		n.d.	-	-	-	-	SPE-LC-MS/MS	[23]

^a^ detection frequency [%]; ^b^ not available; ^c^ not detected; ^d^ limit of quantification; ^e^ semi-quantitative analysis.

**Table 6:** Ecotoxicity data for Z-drugs.

Analyte	Ecotoxicity			Remarks	Ref.
	*Daphnia*	Fish	Algae		
Zolpidem	LC50 ^a^ = 120 mg L^−1^ (48 h)	LC50 = 22 mg L^−1^ (96 h) (*Oncorhynchus mykiss)*	LC50 = 2.2 mg L^−1^ (96 h) NOEC = 0.32 mg L^−1^	From MSDSs	[87]
	LC50 = 1.55 mg L^−1^ (48 h) NOEC ^c^ = 0.019	LC50 = 0.248 mg L^−1^ (96 h) NOEC = 0.023	EC50 ^b^ = 0.211 mg L^−1^ (96 h) NOEC = 0.083	Calculated using ECOSAR software (version 2.2)	[21]
Zolpidem 4-phenylcarboxylic acid	LC50 = 42.8 mg L^−1^ (48 h) NOEC = 0.523	LC50 = 12.4 mg L^−1^ (96 h) NOEC = 0.501	EC50 = 4.69 mg L^−1^ (96 h) NOEC = 1.732	Calculated using ECOSAR software (version 2.2)	[21]

^a^ Lethal Concentration 50%; ^b^ Effective Concentration 50%; ^c^ No Observed Effect Concentration.

**Table 7:** Phytotoxicity data for zolpidem [105].

Morphological Parameters of Seedlings	Physiological Effects on Selected Plant Species
Germination Index (GI)	For *R. sativus*, GI ≈ 100% at 5–15 mg L^−1^; significant decline at 20 mg L^−1^ (GI = 64.2%), indicating phytotoxicity at higher concentration.
Root (Radicle) Growth	*R. sativus*: enhanced radicle elongation at 5 and 15 mg L^−1^ (39 ± 16 mm and 34 ± 10 mm), exceeding control. Suggests stimulation of root development at moderate doses.
Hypocotyl Growth	*R. sativus*: Significant inhibition at 5 and 15 mg L^−1^ (7 ± 3 mm; 9 ± 8 mm), below control values—tendency to promote below-ground over above-ground growth.
Chlorophyll Content (SPAD)	*L. sativa*: Slight increase in SPAD ^a^ at 5 and 10 mg L^−1^ (max = 23.3 SPAD vs. 22.4 control); decline at ≥15 mg L^−1^. *T. aestivum*: Clear SPAD rise at 5 and 10 mg L^−1^ (45.1 and 44.6 SPAD vs. 40.6 control), supporting enhanced photosynthesis.
Shoot Biomass	*L. sativa*: Max = 13.16 ± 2.80 g at 10 mg L^−1^; inhibition at ≥15 mg L^−1^ (≈7.10 ± 1.76 g). *T. aestivum*: Max = 2.60 ± 0.93 g at 10 mg L^−1^; toxicity at 20 mg L^−1^ (1.29 ± 0.47 g).
Root Biomass	*L. sativa*: Highest = 1.59 ± 0.53 g at 10 mg L^−1^; remained > control even at 20 mg L^−1^. *T. aestivum*: Significant increase at 10 mg L^−1^ (1.40 ± 0.42 g).

^a^ SPAD index (Soil Plant Analysis Development).

## Data Availability

No new data were created or analyzed in this study. Data sharing is not applicable.
